# Carbazole Derivatives’ Binding to c-KIT G-Quadruplex DNA

**DOI:** 10.3390/molecules23051134

**Published:** 2018-05-10

**Authors:** Agata Głuszyńska, Bernard Juskowiak, Martyna Kuta-Siejkowska, Marcin Hoffmann, Shozeb Haider

**Affiliations:** 1 Laboratory of Bioanalytical Chemistry, Faculty of Chemistry, Adam Mickiewicz University, Umultowska Street 89b, 61-614 Poznań, Poland; juskowia@amu.edu.pl; 2 Laboratory of Quantum Chemistry, Faculty of Chemistry, Adam Mickiewicz University, Umultowska Street 89b, 61-614 Poznań, Poland; martyna.kuta@o2.pl (M.K.-S.), mmh@amu.edu.pl (M.H.); 3 School of Pharmacy, University College London, London WC1N 1AX, UK; shozeb.haider@ucl.ac.uk

**Keywords:** c-KIT, G-quadruplex, 2O3M, carbazole derivatives, spectroscopy, molecular modeling

## Abstract

The binding affinities of three carbazole derivatives to the intramolecular G-quadruplex (GQ) DNA formed by the sequence 5′-AGGGAGGGCGCTGGGAGGAGGG-3′, derived from the c-KIT 1 oncogene region, were investigated. All carbazole cationic ligands that differed in the substituents on the nitrogen atom were able to stabilize G-quadruplex, as demonstrated using UV-Vis, fluorescence and CD spectroscopic techniques as well as molecular modeling. The spectrophotometric titration results showed spectral features characteristic of these ligands-bathochromic shifts and initial hypochromicity followed by hyperchromicity at higher GQ concentrations. All free carbazole ligands exhibited modest fluorescent properties, but after binding to the DNA the fluorescence intensity increased significantly. The binding affinities of carbazole ligands to the c-KIT 1 DNA were comparable showing values in the order of 10^5^ M^−1^. Molecular modeling highlights the differences in interactions between each particular ligand and studied G-quadruplex, which potentially influenced binding strength. Obtained results relevant that all three investigated ligands have stabilization properties on studied G-quadruplex.

## 1. Introduction

Human genomic DNA contains guanine-rich sequences which can form stable four-stranded structures called G-quadruplexes (GQs) [1]. These tetraplex structures are formed from co-planar arrangements of four guanines that are linked by Hoogsteen-type hydrogen bonds. Resulting GQs are stabilized by stacking interactions between G-tetrads, physiological concentration of monovalent cations (sodium or potassium), and/or by small ligands with characteristic structural features [2,3,4]. The structures of G-quadruplex are important in human telomeres [5], and promoter regions of oncogenes. Due to putative existence of G-quadruplexes in the promoter regions of oncogene such as c-MYC [6,7,8,9], bcl-2 [10,11], RET [12,13], VEGF [14], hTERT [15], or c-KIT [16,17,18], G-quadruplexes have received even more attention after the publication of reports on existence of G-quadruplex structures in human cells [19,20,21]. In the promoter region of oncogene c-KIT, two guanine-rich sequences, known as c-KIT 1 and c-KIT 2, in the KIT promoter upstream from the transcription start site, have been identified [16,17]. The structures of c-KIT G-quadruplex DNA have been determined using the NMR and X-ray crystallography (PDB id: 2O3M [22], 2KQG, 2KQH [23], 2KYP [24], 3QXR [25]). The proto-oncogene c-KIT encodes a receptor tyrosine kinase, which regulates a variety of physiological processes, such as cell proliferation, differentiation, migration, maturation and survival [26,27,28,29]. The overexpression or mutation of c-KIT is identified in many diseases [30], including cancer such as hematopoitic malignancy [31,32,33], prostate [34], adenocarcinoma lung cancers [35], pancreatic cancers [36], seminomas [37], melanoma [38], or gastrointestinal stromal tumors (GIST) [39,40]. In the treatment of GIST, Gleevec^®^ (imatinib mesylate) is used as the kinase inhibitor of c-KIT [41]. The action of this chemical compound directly affects the reduction of c-KIT expression levels, however drug-resistance phenomena were observed [42,43]. Therefore, the synthesis of new molecules as c-KIT G-quadruplex stabilizing ligands that may affect the gene expression, is a promising strategy. There is a growing number of examples of small molecules, capable of inducing and stabilizing GQ formation on c-KIT DNA, for example isoalloxazines [44], bisquinoline-pyrrole oligoamides [45], quinazolones [46], bistriazoles [47,48], acridine derivatives [47,49], alkaloids and their derivatives [50,51,52], oxazole-based peptide macrocycles [53], biaryl polyamides [54], indenoisoquinolines [55] and indenopyrimidines [56], benzo[a]phenoxazines [57], indolylmethyleneindanones [58], bisindole and bisbenzimidazole carboxamides [59,60], terpyridines and their Pt(II) complexes [61], triarylpyridines [62], cationic porphyrins [49,63] and pentaheteroaryls [64], phthalocyanines [65,66], naphthalene diimide derivatives [49,67,68] or anthraquinone and anthracene derivatives [69]. To the best of our knowledge carbazole derivatives, which are highly selective G-quadruplex-binding small molecules [70], have not been examined for interaction with G-quadruplex formed in the c-KIT protooncogene.

Recently, we have reported carbazole derivatives that can specifically stabilize c-MYC G-quadruplex DNA having a parallel topology [71]. Herein, we describe UV-Vis, fluorescence and CD spectra and molecular modeling of three carbazole ligands complexed with c-KIT 1 G-quadruplex. As a template for testing we chose a well-characterized 22-mer G-rich sequence that forms unique parallel-stranded intramolecular G-quadruplex (PDB id 2O3M) [22]. This sequence with or without some modifications has been used frequently as target in the GQ-related experiments.

## 2. Results and Discussion

### 2.1. Ligands and Oligonucleotide

**Figure 1:**
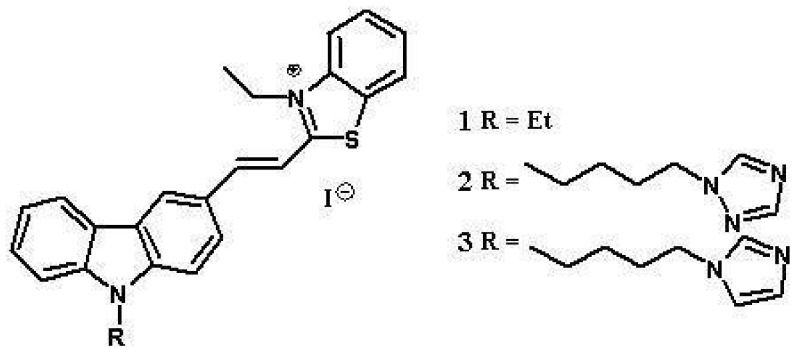
Structures of carbazole ligands **1**–**3**.

Recently, we have been interested in carbazole ligands interacting with G-quadruplexes formed by oligonucleotides with sequences corresponding to promoter regions of oncogenes [71]. The tested compounds **1**–**3** have carbazole skeletons containing a heterocyclic benzothiazolium moiety, a C=C double bond and additional substituents (Figure 1). All these structural elements of the compounds may affect their potential biological properties and influence the stability of c-KIT 1 G-quadruplex [70,71,72,73,74,75,76].

The main structural element that differentiates the three tested compounds is the substituent on the nitrogen atom of carbazole. The replacement of ethyl substituent (ligand **1**) by a more flexible butyl chain terminated with azole ring (triazole in ligand **2** and imidazole in ligand **3**) opens additional opportunities for interactions between the ligands and c-KIT 1 G-quadruplex. Carbazole derivative **1** was synthesized following the published procedure with some modifications [77,78]. Carbazole derivatives **2** and **3** were synthesized by the method reported in our previous paper [74,78]. All the ligand samples were dissolved in DMSO for obtaining stock solution of 1.5 mM concentration and were stored at 4 °C under light protection before use. These compounds are stable for several months, which was controlled using UV-Vis and fluorescence techniques.

**Figure 2:**
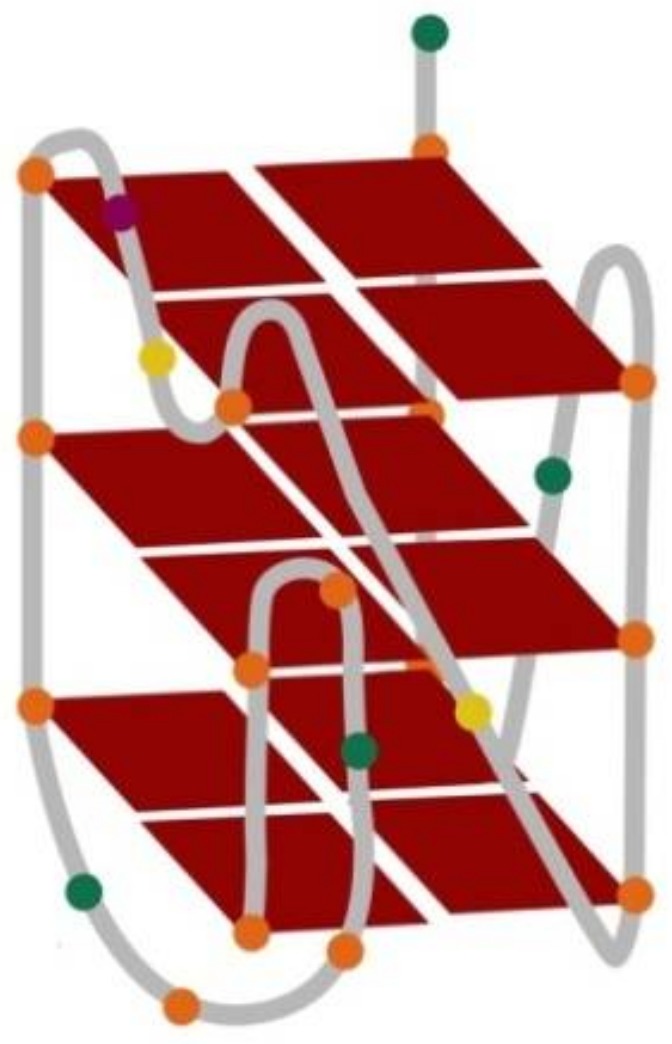
Structure of G-quadruplex formed by c-KIT 1 (5′-AGGGAGGGCGCTGGGAGGAGGG-3′) [PDB id: 2O3M] [22]. Orange ball = guanine, green ball = adenine, yellow ball = cytosine, purple ball = thymine.

As a G-quadruplex scaffold we employed the oligonucleotide bearing the sequence of 5′-A**GGG**A**GGG**C**G**CT**GGG**AGGAG**GG**-3′ found in the promoter region of c-KIT oncogene and called c-KIT 1. This 22-mer G-rich sequence forms unique parallel-stranded intramolecular well-characterized G-quadruplex (PDB id 2O3M) (Figure 2) [22]. This NMR-based structure is composed of three stacked G-tetrads and four connecting loops. One notable and very interesting feature is, that the guanines involved in G-tetrad formation do not come only from G-tracts (in bold, above). Thus, the isolated guanine at position 10 is involved in G-tetrad core formation, despite the presence of four three-guanine tracts-the guanine in position 20 from the last G-tract is excluded. The G-quadruplex 2O3M structure consists of four loops: two single-residue double-chain-reversal loops spanning three G-tetrad layers, a two-residue loop, and a five-residue stem-loop. The base pairings in the loops are important in stabilizing the snapback scaffold of GQ, because the G-tetrad core is interrupted [79].

The 2O3M architecture of c-KIT 1 GQ has been NMR identified for specific conditions: the strand concentration of ca. 5 mM, 20 mM of potassium phosphate (pH 7) containing 70 mM of KCl. The concentration of GQ in binding study is much lower and we have used different media compositions (e.g., Tris-HCl buffer, addition of LiCl), which could affect the specific structural arrangement of 2O3M structure. However, in all experimental conditions used the parallel topology of GQ was evidenced. To evaluate the stabilizing ability and selectivity of ligands **1**–**3** for c-KIT 1 G-quadruplex DNA, the UV-Vis, fluorescence and CD spectroscopic techniques including molecular modeling was carried out.

### 2.2. UV-Vis Absorption Spectroscopy 

**Figure 3:**
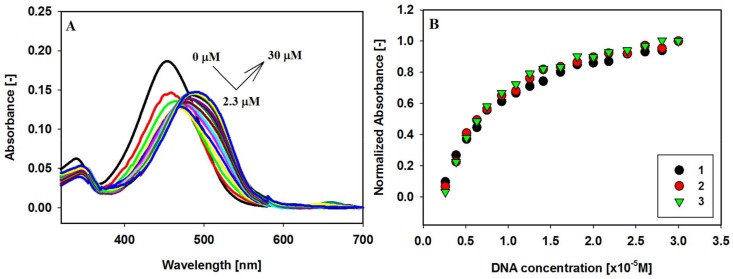
Spectrophotometric titration of ligand **2** (6 µM) with GQ c-KIT 1 (0–30 µM) in Tris-HCl buffer (10 mM, pH 7.2) containing 100 mM KCl (**A**). Panel (**B**) shows normalized absorbance changes vs. increasing concentration of c-KIT 1 G-quadruplex at 515 nm (ligand **1**), 521 nm (ligand **2**), and 508 nm (ligand **3**).

The interaction between the small molecules of carbazole ligands **1**–**3** with G-quadruplex c-KIT 1 was first investigated using visible absorption spectral titrations. The UV-Vis spectra of the free ligands **1**–**3** in the range from 365 to 600 nm exhibit one absorption band centered at about 453 nm, this band can be assigned to the transition within carbazole skeleton conjugated with benzothiazolium system via the double bond C=C (Figure 3A and Figure S1 (Electronic supporting information)). Absorption titration spectra of compounds **1**–**3** with increasing amounts of c-KIT 1 G-quadruplex DNA in 10 mM Tris-HCl buffer containing 100 mM KCl (pH 7.2) are very different from the initial spectrum. Upon addition of GQ DNA, the 453 nm band is red-shifted to 489, 491, and 495 nm (Δλ = 36–42 nm), for ligands **1**, **2** and **3**, respectively. The additional effect observed during these experiments was the initial hypochromicity (30–35%) followed by hyperchromic effect (15–25%) without sharp isosbestic points. The GQ/**1** complex shows the largest decrease in absorbance (hypochromicity 35%), with ligands **2** and **3** complexes showing only slightly less hypochromicity in the absorbance, 30% and 31%, respectively (Table 1). Comparing the data on the bathochromic shift and hypochromic effect, it can be assumed that carbazole ligands may be bound through strong stacking interactions to the G-quadruplex c-KIT 1 structure at the external quartet [80,81]. This is an indication that all tested ligands are stacked on the G-tetrad in the c-KIT 1 G-quadruplex with very similar efficiency.

### 2.3. Fluorescence Spectroscopy

**Figure 4:**
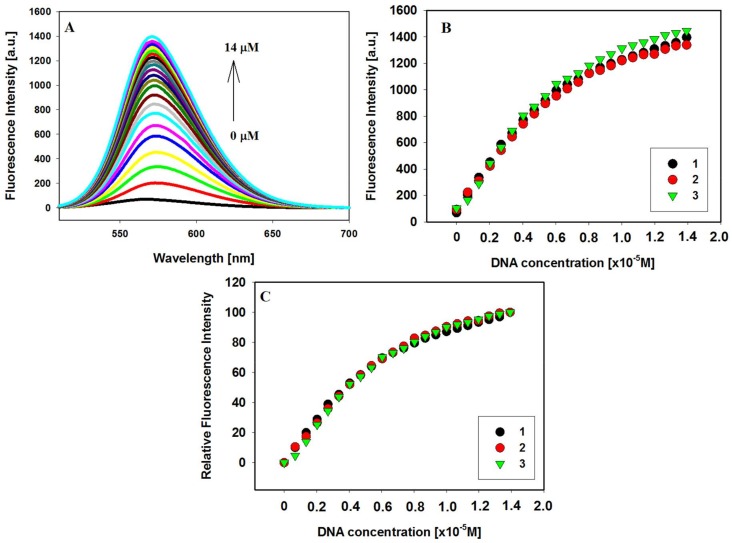
Fluorescence titration spectra of ligand **1** (2 µM) with GQ c-KIT **1** (0–14 µM) in Tris-HCl buffer (10 mM, pH 7.2) containing 100 mM KCl (**A**); Fluorescence binding curves (**B**) and relative enhancement in the fluorescence intensities (**C**) of ligands vs. the increasing concentration of G-quadruplexes c-KIT 1; λ_ex_: **1**,**3**—493 nm, **2**—492 nm.

The binding of ligands to c-KIT 1 G-quadruplex were also evaluated using fluorescence titration. Compounds **1**–**3** are weakly emissive in aqueous Tris HCl/KCl buffer solution. However, the addition of G-quadruplex c-KIT 1 resulted in a significant increase in emission intensity for each ligand. The hydrophobic environment inside the G-quadruplex c-KIT 1 protects the ligand from quenching by polar water molecules and they recover their fluorescence [82,83,84,85]. In accordance with previous observations no plateau was observed for titration plots and the experiments were conducted until the changes in fluorescence spectra were insignificantly small [71]. Figure 4A–C and Figure S2 (ESI) show the fluorescence spectra and binding plots for tested ligands **1**–**3** in the presence of various concentrations of GQ c-KIT 1. The addition of G-quadruplex to solutions of ligands **1**–**3** resulted in an increase in fluorescence intensity of up to 20-fold for ligand **1** and 12–14-fold for ligands **2** and **3**, when the concentration ratio of [GQ]/[L] was increased to 7. However, the final fluorescence intensity after addition this excess of GQ DNA resulted in a very similar fluorescence enhancement (Figure 4B). The maximum emission of free ligand at 566, 567 and 568 nm underwent bathochromic shift after the first additions of G-quadruplex and formation of the complex, by 8 nm, 5 nm and 4 nm for ligands **1**, **2** and **3**, respectively. However, after the addition of 7 eq of GQ only for ligand **1** the bathochromic shift was still observed (4 nm) (Figure 4A). All experiments have shown that relative enhancements in the fluorescence intensities of ligands **1**–**3** were very similar and were correlated with binding constants (Figure 4C, Table 2).

### 2.4. CD Spectroscopy

**Figure 5:**
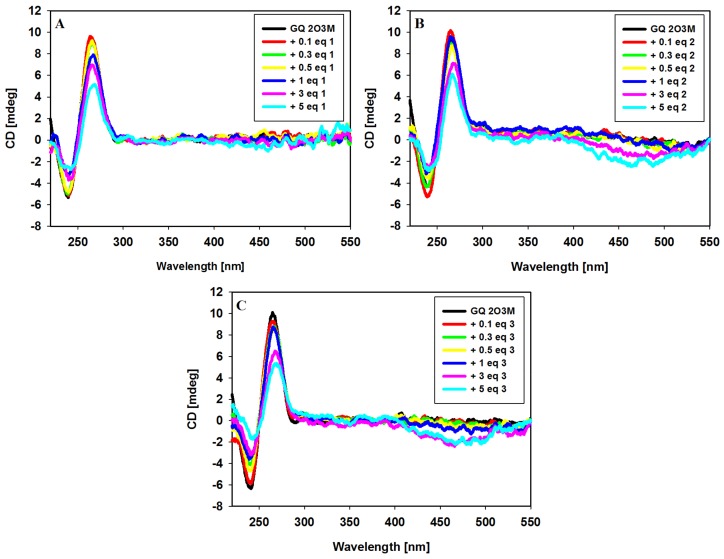
CD spectra of G-quadruplex c-KIT 1 (5µM) with increasing amounts of ligands **1** (**A**), **2** (**B**) and **3** (**C**) in Tris-HCl buffer (10 mM, pH 7.2) containing 100 mM KCl.

We used CD spectroscopy to clarify the effect of three carbazole ligands **1**–**3** on the conformation of c-KIT 1 G-quadruplex. This analytical technique can be used to effectively distinguish between parallel and antiparallel G-quadruplex structures [86]. The CD spectrum of a pre-annealed solution of G-quadruplex c-KIT 1 in a buffer containing 100 mM KCl and 10 mM Tris HCl at pH 7.2 is dominated by a strong positive band at 262 nm and a smaller negative at 245 nm, typical for the parallel G-quadruplex structure [17]. After addition of each portion of ligand, the sample was mixed thoroughly, and the CD spectrum was immediately recorded. In general, addition of ligands did not affect the positions of peaks, suggesting that the G-quadruplex structure retained the parallel topology. Gradual decrease in the intensity of CD signal suggested ligand-dependent perturbation in the perfect stacking between tetrads in the G-quadruplex [87]. Observed deterioration of CD signals at 245 and 262 nm is comparable for all investigated systems (Figure 5), which may suggest similar binding affinities of the ligands. It should be noted that the studied ligands are achiral, so they do not exhibit CD activity in solution. However, one can observe negative induced signals (ICD) in the long-wavelength region where carbazole ligands possess absorption bands. Thus, the CD changes at 450 nm suggest that the G-quadruplex binds ligands similarly, but with varying strength depending on the functional substituents in particular carbazole ligands. These results suggest the same binding modes for all three compounds. Binding of ligand **3** caused much greater increase in ICD peak at the 450 nm than observed for the other two ligands, and the trend of these changes is **3** > **2** > **1,** suggesting similar order of binding affinity for ligands. This c-KIT 1 structure is highly asymmetric and it is preferred to bind to G-tetrad at the 3' end, where the ligand is further stabilized by a five-residue stem-loop [22,31].

### 2.5. Binding of Ligands to c-KIT 1 G-Quadruplex 

The UV-Vis absorption and fluorescence spectral data were analyzed to get the binding constants of ligands **1**–**3** to the c-KIT 1 G-quadruplex DNA, while all spectroscopic methods were used to characterize the ligand binding modes. The UV-Vis and fluorescence titration experiments were performed and the spectral data were quantitatively analyzed by constructing Benesi-Hildebrand (B-H) plots. We used the B-H method to estimate the binding affinities of investigated ligands, because this method still allows data analysis though the binding plot does not reach saturation. The B-H model assumes that the stoichiometry of formed ligand/G4 complex is 1:1 thus the result of B-H calculation should be regard as a *n*K_b_ product (Table 2, Figures S3 and S4 (ESI)) [88]. The G-quadruplex structure formed by 22-mer c-KIT 1 (PDB id 2O3M) is quite unique and assuming that it exists in physiological conditions seems to be a specific target for drug design. The intramolecular G-quadruplex c-KIT 1 (PDB id 2O3M) has two potential ligand binding sites, one at either end of the structure. However, this c-KIT 1 structure is highly asymmetric and the binding to 3′ end is preferred [22,31]. The results indicated that the binding affinities of carbazole ligands to the c-KIT 1 DNA were very comparable and of the order of 10^5^ M^−1^. The binding parameters calculated from absorbance and fluorescence titrations are in good agreement (Table 2). We have determined the binding constant K_b_ for each case on the basis of three independent experiments and the calculated K_b_ values for complexes of ligands **1**–**3** with the c-KIT 1 G-quadruplex were in ranges of 1.1–2.0 × 10^5^ M^−1^ and 1.2–1.5 × 10^5^ M^−1^, for UV-Vis and fluorescence experiments, respectively. Compound **1** with a short side chain showed comparable binding constants (1.1–1.5 ± 0.1–0.2 × 10^5^ M^−1^) obtained for ligands **2** (1.2–1.4 ± 0.1 × 10^5^ M^−1^) and **3** (1.4–2.0 ± 0.1 × 10^5^ M^−1^) with a longer butyl chain additionally terminated with azole rings. It appeared that fluorescence experiments (Figure 4 and Figure S2) supported the external stacking binding modes proposed by the UV-Vis absorption titration results (Figure 3 and Figure S1, Table 1). The fluorescence intensity of weakly emissive ligands **1**–**3** in aqueous buffer solution increased significantly after addition of G-quadruplex c-KIT 1. This suggests that tested ligands are stacked at external quartet of G-quadruplex and to the same extent they are exposed to the solvent (Figure 4B). The spectral shapes of the induced CD signals for all the **1**–**3**/GQ DNA complexes were similar, suggesting that the carbazoles should interact with G4 DNA at the same binding site but with different affinity since intensities of these ICD bands were not the same.

### 2.6. DNA Melting Studies

**Figure 6:**
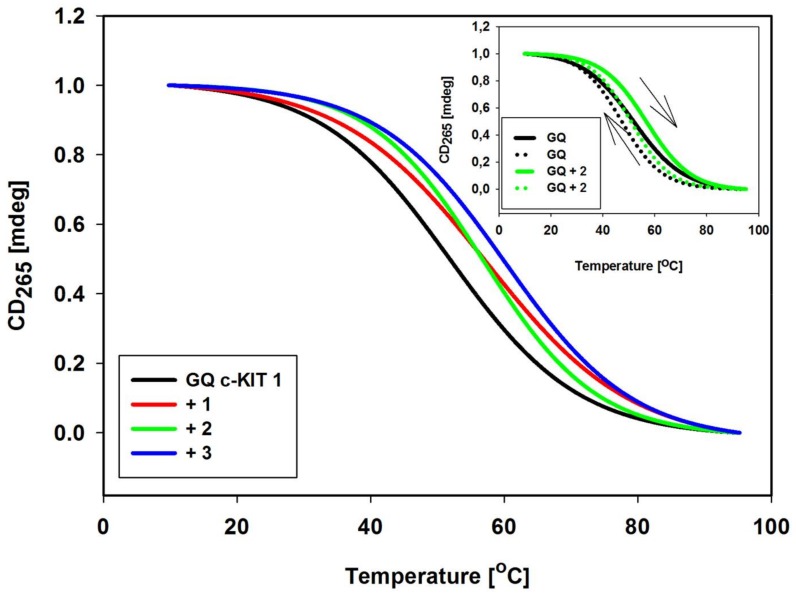
Normalized CD melting profiles of c-KIT 1 G-quadruplex at 265 nm with and without 3 eq of ligands **1**–**3** in 10 mM Tris-HCl buffer (pH 7.2) containing 90 mM LiCl and 10 mM KCl. Inset: The effect of rates of heating and cooling on CD profiles of GQ c-KIT 1 in absence and presence of ligand 2. Solid lines are melting curves, dotted lines are annealing curves.

To determine thermal stability of GQ c-KIT 1 DNA formed in the presence of each carbazole ligand **1**–**3**, a CD melting study was performed in 10 mM Tris-HCl buffer (pH 7.2) containing 100 mM KCl, as well as in low ionic strength buffer (K^+^ concentration reduced to 10 mM and addition of 90 mM LiCl) [89]. The T_m_ of GQ in the absence of ligand in 100 mM KCl solution was high and amounted to 77.9 °C. Reduction of the amount of potassium, as we expected, resulted in a lowering of T_m_ of unbound GQ to 51.8 °C and in these conditions changes in T_m_ (ΔT_m_ values) in the presence of ligands **1**–**3** at 1:3 molar ratio (GQ:ligand) were 5.9, 5.4, and 8.4 °C, respectively (Table 3, Figure 6).

The hysteresis between the melting and annealing curves (1 °C min^−1^ rates of temperature change) was observed (denaturation curves were shifted toward higher temperatures than renaturation curves), indicating the slow kinetics (Figure 6) [90,91]. The tested ligands showed comparable intermediate level of stabilization on GQ c-KIT 1. These data are in good agreement with those obtained by UV-Vis absorption and fluorescence measurements.

### 2.7. Comparison with c-MYC G-Quadruplex

We have been interested to check the effect of carbazole ligands on stabilization of promoter G-quadruplex DNAs c-MYC and c-KIT [71]. UV-Vis and fluorescence experiments were conducted to find spectral effects for the ligands **1**–**3** bound to GQs DNA and the binding constants of the ligands with c-MYC and c-KIT GQs. In any case ligands exhibit the bathochromic shift, hypochromic and hyperchromic effect in UV-Vis titration experiments and increase in the fluorescence intensity in fluorescence titration experiments after addition of GQ. The addition of the c-MYC G-quadruplex to solution of ligands resulted in a red-shift by 20 nm higher, compared to analogous experiments with c-KIT G-quadruplex, as well as the binding constants of carbazole ligands with c-MYC GQ were found to be 3.9–6.9 ± 0.1 × 10^5^ M^−1^ , which are ~3–4 fold higher than these of c-KIT GQ (Table 4).

**Figure 7:**
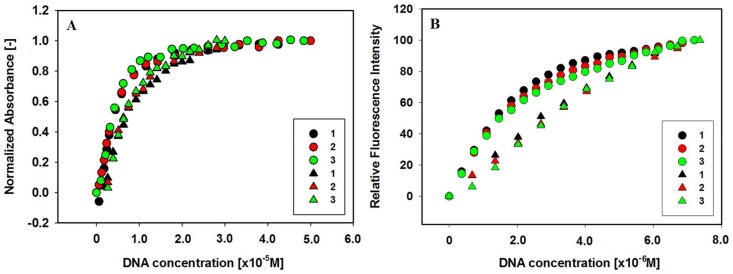
(**A**) Normalized absorbance changes vs. increasing concentration of G-quadruplexes c-MYC (circles) and c-KIT 1 (triangles) for ligands **1**, **2** and **3**. (**B**) Relative enhancement in the fluorescence intensities of ligands vs. the increasing concentration of G-quadruplexes c-MYC (circles) and c-KIT 1 (triangles). Color assignment: ligand **1** (black), ligand **2** (red), and ligand **3** (green).

All experiments and calculations carried out showed stronger interaction of the tested ligands with c-MYC G-quadruplex structure (Figure 7, Table 4).

Analyzing the experimental results, we can see that the substituent attached to the nitrogen atom of carbazole skeleton (an azole rings against aliphatic ethyl chain) does not have a major impact on the stability of the complex ligand-GQ, however, substituent may have an effect on the biological activity of the compounds [70].

Tested GQ are known to adopt parallel-stranded folding topology in K^+^ solution. However, these structures are probably very different from each other—the c-MYC G-quadruplex possesses three double-chain-reversal loops, while the G-quadruplex of c-KIT 1 contains four loops: two single-residue double-chain-reversal loops spanning three G-tetrad layers, a two-residue loop, and a five-residue stem-loop. In our opinion, in these differences one should see the difference in the strength of interactions.

### 2.8. Molecular Modeling Studies

**Figure 8:**
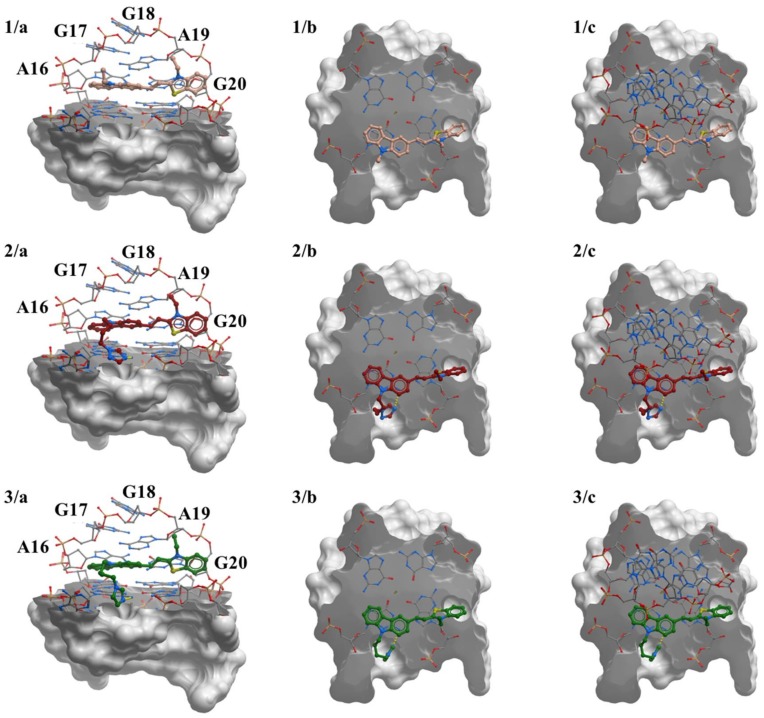
Docked ligands **1**, **2** and **3** on external G-tetrads of parallel-stranded G-quadruplex (PDB id 2O3M). Cartoons representing (**a**) the side view of the complex. In (**a**), the 3′ terminal quartet is illustrated as sticks and the 5′ end in a surface representation. The next two illustrations highlight (**b**) the top view of the 3′ quartet (without the loop) and (**c**) the 3′ quartet with the loop. Hydrogen bonds are highlighted as yellow dotted lines.

All ligands **1**–**3** were docked into the cleft at the 3′ end of G-quadruplex (tetrad G4-G8-G15-G22). Docking results for all ligands showed that the most significant interaction between researched ligands and c-KIT 1 quadruplex was end-stacking interactions (Figure 8).

In each case we observed the carbazole moiety with benzothiazolium lying on the exposed G4 and G8 residues. The deflection (twisting) of the benzothiazolium moiety is observed in each case. This is due to interactions with the loop above G-tetrad (A16-G17-G18-A19-G20). Ligand **1** does not make any interactions with the groove since it does not possess any side chain. In contrast, for ligand **2** and **3** complexes, the triazole or imidazole groups are placed in the groove between residues A5 and C9. Both imidazole and triazole make one hydrogen bond with nitrogen from external guanine from G4 (Figure 8 highlighted as a yellow dotted line).

From the simulations (Figure S5), we assessed how the ligands interacted with the 2O3M quadruplex. During the course of the simulation, ligand **1** did not translate on the quartet, which meant that the carbazole interacted by strong end-stacking interactions with the G4. The double bond between carbazole moiety and benzothiazolium stayed in the trans conformation indicating that flipping of the benzothiazolium moiety was not observed. In comparison with the conformation of the other docked ligands, we observed only a small shift of ligand **1** in the binding site, suggesting end-stacking interaction by the entire ligand surface. Ligand **2** shifted above external G-tetrad (binding pocket), when compared with the position of ligand **1**. As shown in Figure S5B (ESI), the initial position of the ligand **2** (green structure) represents the conformation after 200 ns. The carbazole skeleton with benzothiazolium is almost flat and interacts with G-tetrad by end-stacking interaction while the side chain with triazole is moved from groove between A5-C9 residues and is positioned near the long loop A16-G17-G18-A19-G20. After 400 ns simulation (pink structure) no change in ligand position was observed. After 600 ns of the simulation, we observe that ligand **2** changed the position completely. The benzothiazolium moiety is directed towards the groove created by backbone of the G4 and G15 while triazole is placed in the groove between residue A5 and C9. After 800 ns, the position of ligand **2** changes again. The carbazole with a benzothiazolium moiety interacts by end-stacking interactions with the external G-tetrad. The benzothiazolium moiety overlaps the tetrad and the carbazole group covers G8 nucleotide. After 1000 ns, the conformation and position of the carbazole with benzothiazolium is stable while the side chain with triazole is more flexible. Similarly, ligand **3** exhibited noticeable mobility in the binding pocket on external tetrad (Figure S5C).

**Figure 9:**
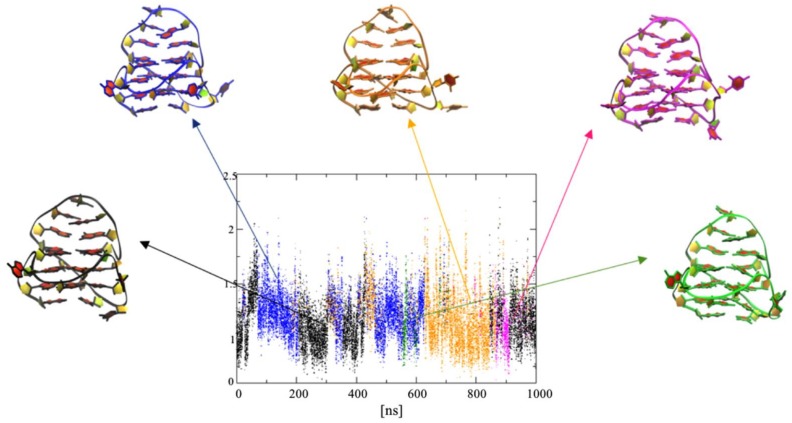
Representation of the conformational clusters obtained by clustering analysis for G-quadruplex-ligand **3** complex. RMSD-based clustering, with a cut-off of 2.3 Å, identified five clusters. Cartoon representations of side view of cluster centers are illustrated (top 3′ end).

Analysis of the RMSD values indicates that our systems are indeed well equilibrated at the current timescale (Figures S6 and S7). No significant changes in RMSD were observed, which suggest similar stabilizing properties for the ligands. Calculated RMSF value (root mean square fluctuation) is a good indicator to show the intensity of fluctuation of each nucleotide throughout the simulations. The results show that A19 is stabilized when it interacts with ligand **1** (Figure S8 and Figure 9). Binding of ligand **1** also has influence on the G-tetrad on the 5′end of G-quadruplex, lowering the RMSF values of all tetrad-forming residues. Obtained results revealed that both ligand **2** and **3** also stabilize A19. Unfortunately, they do not reduce the value of RMSF for residues 9 to 11 from long loop. This might be due to the presence and flexibility of the side chain arm present in ligand **2** and **3**. In order to understand how the ligands bind to c-KIT 1 structure and how they influence the conformation of the quadruplex, we carried out extensive clustering analysis. The aim of clustering analysis is to identify the main sub-states sampled during the course of the simulation. In this section we present result for ligand **3** since experiment of melting temperature revealed that this ligand has best stabilization properties of studied GQ.

The most significant differences between clusters are observed for loop C9-G10-C11 and residue A5 (Figure S12). A 2.3 Å RMSD cut-off identified 2 cluster conformations for complex of G4 simulated with ligand **1** (Figure 9). The most significant differences between clusters are observed for loop C9-G10-C11 and residue A5 (Figure S12). A 2.3 Å RMSD cut-off identified 5 cluster conformations for complex of G4 simulated with ligand **3** (Figure 8). Most significant clusters are: black (34.4%), blue (33%), and orange (27.1%). The transition centroids, which represent clusters magenta (3.7%) and green (1.9%) are small. Cluster analysis with 2.3 Å RMSD cut-off for ligand **1** complex identifies 2 clusters (Figure S13). The first 200 ns were represented by the blue cluster, which comprised around 20% of entire simulation. The second cluster (black) represented ~80% of entire simulation. Differences that took effect on the separation of two clusters result from conformation changes of residue C11 in the loop C9-G10-C11 and residue A5 (Figure S14). Cluster analysis with 2.3 Å RMSD cut-off for ligand **2** complex identifies 3 clusters (Figure S15). For the first 500 ns simulations we observed centroid orange (~34%), followed by a transition state represented by centroid blue (~14%). After 500 ns, the black cluster (~51%) dominates with the transition to the conformation represented by the orange centroid. Similarly, as for other complexes we identified that changes in the conformation of the simulated quadruplex was being generated from the conformational changes in loop C9-G10-C11 and residue A5 (Figure S16).

## 3. Materials and Methods 

### 3.1. Ligands

Carbazole derivative **1** was synthesized following the published procedure with some modifications [77,78]. Carbazole derivatives **2** and **3** were synthesized by the method reported in our previous papers [71,74]. The purity of ligands was examined by the HPLC technique. These studies were performed on a Waters HPLC system 1525 (Waters Corporation, Milfort, MA, USA) equipped with a mod. 2998 Photodiode Array detector and a mod. 2475 fluorescence detector, and a Breeze interface. A Symmetry®C18 column (d_p_ = 3.5 μm, 75 × 4.6 mm i.d.) Waterswas used with isocratic elution (70% MeOH and 30% 10 mM NaCl, flow rate 1 mL·min^−1^).

### 3.2. Oligonucleotide

The quadruplex-forming 22-mer deoxyribonucleotide with c-KIT sequence of 5′-AGGGAGGGCGCTGGGAGGAGGG-3′ (2O3M code in the Protein Data Bank) was purchased from Genomed (Poland) and was used without further purification. The strand concentration was determined at 260 nm at 85 °C using extinction coefficient of 257,600 M^−1^ cm^−1^ as calculated from the published values of molar absorptivities of nucleotides [92]. Before using, the oligonucleotide solution was heated at 90 °C for 5 min and subsequently allowed to slow cooling to room temperature, and stored at 4 °C overnight. Tris Base (CAS Number 77-86-1) and Tris HCl (CAS Number 1185-53-1) were obtained from Aldrich Chemical Co. (Poznań, Poland) and used as received.

### 3.3. Absorption Spectroscopy

The absorption spectra were recorded on a Cecil CE-2021 spectrophotometer in the 200–700 nm range at 25 °C. All of the measurements were carried out using a 10 mm quartz cell. UV-Vis absorption titrations were carried out by the stepwise addition of small aliquots of 766.8 µM/strand of G4 DNA 2O3M solution to a cell containing 1000 µL of 6 µM ligand in buffer. Three min was the equilibration time after each DNA addition. All measurements were performed in a 10 mM Tris-HCl buffer (pH 7.2) containing 100 mM KCl.

### 3.4. Fluorescence Spectroscopy

The fluorescence measurements were carried out using a FP 8200 spectrofluorimeter (Jasco, Tokyo, Japan). The cell compartments were thermostated at 25 °C. All of the measurements were carried out using a 10 mm quartz cell. The fluorescence spectra were collected from 510 to 750 nm with both excitation and emission slits being 5 nm. Fluorescence titrations were carried out by the stepwise addition of small portions of 677 µM/strand of G4 DNA 2O3M solution to a cell containing 1000 µL of 2 µM ligand in buffer. Three min was the equilibration time after each DNA addition followed by emission spectrum recording. Excitation wavelength depended on the absorption spectrum of the ligand. All measurements were performed in a 10 mM Tris-HCl buffer (pH 7.2) containing 100 mM KCl.

### 3.5. Circular Dichroism 

Circular dichroism (CD) spectra were recorded on a Jasco J-810 spectropolarimeter in the spectral range from 210 to 600 nm with 500 nm/min scan speed and bandwidth of 1 nm. Spectra were recorded in quartz cuvettes of 1 cm path length and averaged from 3 scans. Measurements with oligonucleotide were performed at 25 °C in a 10 mM Tris-HCl buffer (pH 7.2) containing 100 mM KCl. Concentration of 22-mer oligonucleotide 2O3M was 5 µM/strand. Ligands were added to G4 DNA solution at increasing concentration from 0.1 to 5 mol equivalents.

In the melting studies, the temperature of the samples were maintained by a Jasco Peltier temperature controlled cell holder. The melting profiles of the c-KIT G-quadruplex samples were prepared by heating the 2 μM oligonucleotide solution in 10 mM Tris HCl buffer, pH 7.2, and 90 mM LiCl and 10 mM KCl at 90 °C for 5 min followed by slow cooling, and storing at 4 °C overnight. The melting profiles were recorded in the absence and presence of 3 eq of ligands **1**–**3** in 10–95 °C range with a 1 °C/min scan speed. All experiments were carried out using quartz cuvettes with a 10 mm optical path. Data were collected at 265 nm. Typically three replicate experiments were performed, and average values are reported.

### 3.6. Ligand-Quadruplex Binding Study

Binding data obtained from spectrophotometric and spectrofluorimetric titrations were analyzed according to Benesi-Hildebrand transformation [88]. Experiments were carried out in the same manner-after each G4 DNA addition, the titrated solution was incubated for 3 min followed by the UV-Vis or fluorescence spectrum measurement. The titration was continued until only small changes in the absorption or fluorescence spectra were observed upon successive addition of 2O3M.

The Benesi-Hildebrand transformation used to estimate the value of K_b_ is represented by the Equations (1) and (2) that describe the one-site ligand binding model:

(1) $$\frac{\mathrm{cL}}{A-\mathrm{Ao}}\;=\;\frac{\mathrm{cL}}{\mathrm{Am}-\mathrm{Ao}}\;+\;\frac{\mathrm{cL}}{\left(\mathrm{Am}-\mathrm{Ao}\right)n\mathrm{Kb}}\times \;\frac{1}{\mathrm{cG}4\;\mathrm{DNA}}$$

(2) $$\frac{1}{F-\mathrm{Fo}}=\;\frac{1}{\mathrm{Fm}-\mathrm{Fo}}\;+\;\frac{1}{\left(\mathrm{Fm}-\mathrm{Fo}\right)n\mathrm{Kb}}\;\times \;\frac{1}{\mathrm{cG}4\;\mathrm{DNA}}$$

where cL is total concentration of ligand, Ao is the absorbance of ligand in the absence of G4 DNA, A is the absorbance recorded in the presence of added G4 DNA at the concentration of cG4 DNA, Am is absorbance in presence of added [cG4 DNA]max, Fo is the fluorescence of ligand in the absence of G4 DNA, F is the fluorescence recorded in the presence of added G4 DNA, Fm is fluorescence in presence of added [cG4 DNA]max, and n is the number of bound ligand molecules per G-quadruplex, K_b_ is the binding constant.

### 3.7. Molecular Modeling Studies

The design protocol was based on the combined use of docking and molecular dynamics simulations, in which the ligand, the target and the solvent were described by an atomistic force field. In particular, we first used the docking calculations in order to establish the favourable position of the ligands. We then performed molecular dynamics simulations to check the stability of studied complexes. The final quality measure was the average value of the scoring function on a long molecular dynamics simulation. The sequence from c-KIT 1 (PDB ID: 2O3M, 5′-(1A-2G-3G-4G-5A-6G-7G-8G-9C-10G-11C-12T-13G-14G-15G-16A-17G-18G-19A-20G-21G-22G)-3′ promoter region was chosen as a model for G-quadruplex for both molecular docking of the ligands and molecular simulations [22]. We used the DNA modifications to AMBER force field for system modeling [93,94,95,96,97]. The topologies were generated by tleap program in the AMBERTOOLS software package [98].

### 3.8. Ligand Docking and Modeling

Parallel G-quadruplex (PDB id: 2O3M) formed on c-KIT 1 promoter region was used as a receptor in docking **1**–**3** ligands. Docking grid was centred on 3′ end of the terminal G4, specifically on G4-G8-G15-G22 G-tetrad and the long loop (A16-G17-G18-A19-G20). The G-quadruplex and K^+^ ions were kept frozen in the original conformation throughout the docking procedures. For each ligand we used the default values of docking in ICM Molsoft program [99]. Best position of the ligand was chosen based on the binding score.

### 3.9. Molecular Dynamics Simulations

The solution structure of monomeric parallel-stranded G-quadruplex formed from c-KIT 1 gene promoter region 5′-(AGGGAGGGCGCTGGGAGGAGGG)-3′ (PDB id: 2O3M) was used as the starting structure for the simulation. 2 K^+^ ions were added in the central channel between the quartets. The structure was explicitly solvated with TIP3PBOX water molecules in a periodic box whose boundaries extended at least 10 Å from any solute atom. Additional K^+^ counter ions were added to the system to neutralize the charge on the quadruplex backbone. The counterions were automatically placed, using the Coulombic potential, into the most negative locations by the LEAP program. Periodic boundary conditions were applied to avoid the edge effect. The particle mesh Ewald (PME) method was employed to calculate long-range electrostatic interactions. The AMBER ff99sb force field [94] with parmbsc1 [95] and χ_OL3+OL15_ [96,97] modifications were applied for G-quadruplexes, ions, and water molecules. The ligands were parameterised using the GAFF force field [93]. A 10 ns MD equilibration was performed, in which system was restrained while solvent and ions were allowed to equilibrate. The production run with no restraints on the system was run for 1 μs (1000 ns) simulations using the ACEMD molecular dynamics engine [100]. The trajectories were analyzed using the g_rmsd, g_rmsf and g_sas module available in the GROMACS suite (www.gromacs.org) [101], and visualized by means of the VMD program [102,103] and ICM-Pro Molsoft molecular modelling package [99].

### 3.10. Interactions and Stability Analysis

To assess the stability of the complexes we calculated root-mean-square-deviation (RMSD) and root-mean-square fluctuation (RMSF) values [104]. The result allows the evaluation of the conformational flexibility of the loops and G-tetrad and checks the influence of the ligands on stabilisation of the quadruplex structure.

### 3.11. Clustering

Clustering is a useful tool to group members of structure, which are similar to each other into clusters. To identify the number of structure into clusters it is necessary to use differentiating parameters. In our protocol we used RMSD (root mean square deviation) to identify structural clusters from a trajectory. RMSD parameter enables to calculate the pairwise distances measured as coordinates between structures are defined by a cut-off value reflecting the range of conformations and their relative populations. The algorithm generates the most representative centroids structure for each cluster and then gives an RMSD for each structure in the trajectory with respect to each identified cluster. The MMTSB toolkit code was used with a cut-off of 2.3 Å for all frames from trajectory, which were extracted at a time interval of 40 ps for a total of 25 000 frames [95].

## 4. Conclusions

In summary, three carbazole compounds were investigated as c-KIT 1 G-quadruplex targeting probes. The ligand-quadruplex complexes have been evaluated by UV-Vis spectrophotometry, fluorescence, CD spectroscopy and molecular modeling. Our experimental results have shown that introduction of different substituents on the nitrogen atom of carbazole exerted modest impact on the G-quadruplex binding properties, demonstrating similar stabilization of GQ and the very comparable binding affinities in the order of 10^5^ M^−1^. Large spectral changes in ligands spectra at higher G4 concentrations have been observed-the bathochromic shifts and initial hypochromicity in UV-Vis absorption spectra as well as large fluorescence enhancement. All these suggested the end-stacking binding mode of carbazole ligands on the external G-tetrad c-KIT 1 quadruplex. Molecular modeling calculations revealed also the end stacking binding mode, but in the case of ligands **2** and **3**, it was reinforced by hydrogen bonding between tethered imidazole or triazole and the floor of G-quadruplex groove. Our experimental results have not proved higher binding affinity of ligand **2** and **3** comparing to ligand **1**, which may suggest rather weak hydrogen bonding in relation to stronger end stacking interactions. Alternative explanation may involve differences in 2O3M structure adapted for the molecular calculations and the actual structure of G-quadruplex formed by c-KIT 1 in experimental conditions used in this work.

## Figures and Tables

**Table 1:** Spectral effects for the ligands **1**–**3** bound to c-KIT 1 GQ DNA.

Ligands	Δλ_max_ (nm) ^a^	Hypochromicity (%) ^b^	Hyperchromicity (%) ^b^
1	36	35	15
2	38	30	16
3	42	31	25

^a^ Δλ bathochromic shift. ^b^ Hypochromicity and hyperchromicity were measured at λ_max_. The hypo- and hyperchromicity percentages were calculated using following equations: %Hypo = [(A_f_ − A_b,min_)/A_f_] × 100 and %Hyper= [(A_b,max_ − A_b,min_)/A_b,max_] × 100, where A_f_, A_b,min_, A_b,max_ are the absorbances of free ligand, bound ligand with absorbance minimum, and bound ligand with absorbance maximum (excess of GQ), respectively.

**Table 2:** Parameters for the interaction of ligands **1**, **2** and **3** with c-KIT 1 G-quadruplex determined using Benesi-Hildebrand method in absorption and fluorescence titration experiments (K_b_—binding constant, *n*—number of bound ligand molecules per G-quadruplex).

Ligands	Benesi-Hildebrand Method, *n*K_b_ (× 10^5^ M^−1^)	
	Spectrophotometric Titration	Fluorescence Titration
1	1.1 ± 0.1	1.5 ± 0.2
2	1.4 ± 0.1	1.2 ± 0.1
3	2.0 ± 0.1	1.4 ± 0.1

**Table 3:** Summary of the DNA melting studies.

Derivative	T_m_ (°C) ^a^	ΔT_m_ (°C) ^b^	T_m_ (°C) ^c^	ΔT_m_ (°C) ^b^
No drug	51.8	-	77.9	-
1	57.7	5.9	79.9	2.0
2	57.2	5.4	79.9	2.0
3	60.2	8.4	82.2	4.3

^a^ T_m_ of GQ c-KIT 1 in the absence and presence of ligands **1**–**3** in 10 mM Tris-HCl buffer (pH 7.2) in the presence of 90 mM LiCl and 10 mM KCl. ^b^ ΔT_m_ were obtained from the differences in the melting temperatures of the 3 eq ligand bound and uncomplexed GQ DNA. Data were collected at 265 nm. Typically three replicate experiments were performed, and average values are reported within ±1 °C of each other. ^c^ T_m_ of GQ c-KIT 1 in the absence and presence of ligands **1**–**3** in 10 mM Tris-HCl buffer (pH 7.2) containing 100 mM KCl.

**Table 4:** Spectral effects and parameters for the interaction of ligands **1**, **2** and **3** with c-MYC and c-KIT 1 G-quadruplexes determined using Benesi-Hildebrand method and data from fluorescence titration experiments (K_b_—binding constant, *n*—number of bound ligand molecules per G-quadruplex).

Effect	Ligands	c-MYC ^a^	c-KIT
Δλ_max_ (nm) ^b^	**1**	57	36
	**2**	52	38
	**3**	52	42
Hypochromicity (%) ^c^	**1**	30	35
	**2**	24	30
	**3**	28	31
Hyperchromicity (%) ^c^	**1**	32	15
	**2**	14	16
	**3**	18	25
Fluorescence titration	**1**	6.9 ± 0.1	1.5 ± 0.2
	**2**	4.0 ± 0.1	1.2 ± 0.1
	**3**	3.9 ± 0.1	1.4 ± 0.1

^a^ [71]. ^b^ Δλ bathochromic shift. ^c^ Hypochromicity and hyperchromicity were measured at λ_max_. The hypo- and hyperchromicity percentages were calculated using following equations: %Hypo = [(A_f_ − A_b,min_)/A_f_] × 100 and %Hyper = [(A_b,max_ − A_b,min_)/A_b,max_] × 100, where A_f_, A_b,min_, A_b,max_ are the absorbances of free ligand, bound ligand with absorbance minimum, and bound ligand with absorbance maximum (excess of GQ), respectively.

## Supplementary Materials

The following are available online, Figure S1: Spectrophotometric titration of ligands with G4 c-KIT 2O3M. Figure S2: Fluorescence titration spectra of ligands with G4 c-KIT 2O3M. Figures S3 and S4: Benesi-Hildebrand plots for absorbance and fluorescence binding data of ligands with G4 c-KIT 2O3M. Figures S5–S16: Molecular modeling results.

## References

[1] Burge S., Parkinson G.N., Hazel P., Todd A.K., Neidle S. (2006). Quadruplex DNA: Sequence, topology and structure. Nucleic Acids Res..

[2] De Cian A., Lacroix L., Douarre C., Temime-Smaali N., Trentesaux C., Riou J.F., Mergny J.L. (2008). Targeting telomeres and telomerase. Biochimie.

[3] Ou T.M., Lu Y.J., Tan J.H., Huang Z.S., Wong K.Y., Gu L.Q. (2008). G-quadruplexes: targets in anticancer drug design. Chem. Med. Chem..

[4] Folini M., Gandellini P., Zaffaroni N. (2009). Targeting the telosome: Therapeutic implications. Biochim. Biophys. Acta.

[5] Parkinson G.N., Lee M.P.H., Neidle S. (2002). Crystal structure of parallel quadruplexes from human telomeric DNA. Nature.

[6] Mathad R.I., Hatzakis E., Dai J., Yang D. (2011). c-MYC promoter G-quadruplex formed at the 5′-end of NHE III_1_ element: Insights into biological relevance and parallel-stranded G-quadruplex stability. Nucleic Acids Res..

[7] Dai J., Carver M., Hurley L.H., Yang D. (2011). Solution Structure of a 2:1 Quindolinec-MYC G-Quadruplex: Insights into G-Quadruplex-Interactive Small Molecule Drug Design. J. Am. Chem. Soc..

[8] Phan A.T., Modi Y.S., Patel D.J. (2004). Propeller-Type Parallel-Stranded G-Quadruplexes in the Human c-myc Promoter. J. Am. Chem. Soc..

[9] Simonsson T., Pecinka P., Kubista M. (1998). DNA tetraplex formation in the control region of c-myc. Nucleic Acids Res..

[10] Dexheimer T.S., Sun D., Hurley L.H. (2006). Deconvoluting the Structural and Drug-Recognition Complexity of the G-Quadruplex-Forming Region Upstream of the *bcl-2* P1 Promoter. J. Am. Chem. Soc..

[11] Agrawal P., Lin C., Mathad R.I., Carver M., Yang D. (2014). The Major G-Quadruplex Formed in the Human BCL-2 Proximal Promoter Adopts a Parallel Structure with a 13-nt Loop in K^+^ Solution. J. Am. Chem. Soc..

[12] Guo K., Pourpak A., Beetz-Rogers K., Gokhale V., Sun D., Hurley L.H. (2007). Formation of Pseudosymmetrical G-Quadruplex and i-Motif Structures in the Proximal Promoter Region of the *RET* Oncogene. J. Am. Chem. Soc..

[13] Tong X., Lan W., Zhang X., Wu H., Liu M., Cao C. (2011). Solution structure of all parallel G-quadruplex formed by the oncogene *RET* promoter sequence. Nucleic Acids Res..

[14] Agrawal P., Hatzakis E., Guo K., Carver M., Yang D. (2013). Solution structure of the major G-quadruplex formed in the human VEGF promoter in K^+^: Insights into loop interactions of the parallel G-quadruplexes. Nucleic Acids Res..

[15] Lim K.W., Lacroix L., Yue D.J.E., Lim J.K.C., Lim J.M.W., Phan A.T. (2010). Coexistence of two distinct G-quadruplex conformations in the hTERT promoter. J. Am. Chem. Soc..

[16] Rankin S., Reszka A.P., Huppert J., Zloh M., Parkinson G.N., Todd A.K., Ladame S., Balasubramanian S., Neidle S. (2005). Putative DNA Quadruplex Formation within the Human *c-kit* Oncogene. J. Am. Chem. Soc..

[17] Fernando H., Reszka A.P., Huppert J., Ladame S., Rankin S., Venkitaraman A.R., Neidle S., Balasubramanian S. (2006). A Conserved Quadruplex Motif Located in a Transcription Activation Site of the Human c-kit Oncogene. Biochemistry.

[18] Todd A.K., Haider S.M., Parkinson G.N., Neidle S. (2007). Sequence occurrence and structural uniqueness of a G-quadruplex in the human c-kit promoter. Nucleic Acids Res..

[19] Biffi G., Tannahill D., McCafferty J., Balasubramanian S. (2013). Quantitative visualization of DNA G-quadruplex structures in human cells. Nat. Chem..

[20] Biffi G., Di Antonio M., Tannahill D., Balasubramanian S. (2014). Visualization and selective chemical targeting of RNA Gquadruplex structures in the cytoplasm of human cells. Nat. Chem..

[21] Murat P., Balasubramanian S. (2014). Existence and consequences of G-quadruplex structures in DNA. Curr. Opin. Genet. Dev..

[22] Phan A.T., Kuryavyi V.V., Burge S., Neidle S., Patel D.J. (2007). Structure of an unprecedented G-quadruplex scaffold in the human c-kit promoter. J. Am. Chem. Soc..

[23] Hsu S.T.D., Varnai P., Bugaut A., Reszka A.P., Neidle S., Balasubramanian S. (2009). A G-rich sequence within the c-kit oncogene promoter forms a parallel G-quadruplex having asymmetric G-tetrad dynamics. J. Am. Chem. Soc..

[24] Kuryavyi V., Phan A.T., Patel D.J. (2010). Solution structures of all parallel-stranded monomeric and dimeric G-quadruplex scaffolds of the human c-kit2 promoter. Nucleic Acids Res..

[25] Wei D., Parkinson G.N., Reszka A.P., Neidle S. (2012). Crystal structure of a *c-kit* promoter quadruplex reveals the structural role of metal ions and water molecules in maintaining loop conformation. Nucleic Acids Res..

[26] Yarden Y., Kuang W.J., Yang F.T., Coussens L., Munemitsu S., Dull T.J., Chen E., Schlessinger J., Francke U., Ullrich A. (1987). Human proto-oncogene c-kit: A new cell surface receptor tyrosine kinase for an unidentified ligand. EMBO J..

[27] Roskoski R.J. (2005). Structure and regulation of Kit protein-tyrosine kinase, the stem cell factor receptor. Biochem. Biophys. Res. Commun..

[28] Edling C.E., Hallberg B. (2007). c-Kit—A hematopoietic cell essential receptor tyrosine kinase. Int. J. Biochem. Cell Biol..

[29] Gregory B.E., Bartlett E., Kiupel M., Hayes S., Yuzbasiyan G.V. (2010). Canine and human gastrointestinal stromal tumors display similar mutations in c-KIT exon 11. BMC Cancer.

[30] Lennartsson J., Rönnstrand L. (2012). Stem cell factor receptor/c-kit: from basic science to clinical implications. Physiol Rev..

[31] Wang Y.Y., Zhou G.B., Yin T., Chen B., Shi J.Y., Liang W.X., Jin X.L., You J.H., Yang G., Shen Z.X. (2005). AML1-ETO and C-KIT mutation/overexpression in t(8;21) leukemia: Implication in stepwise leukemogenesis and response to Gleevec. J. Proc. Natl. Acad. Sci. USA.

[32] Ferrari S., Grande A., Zucchini P., Manfredini R., Tagliafico E., Rossi E., Temperani P., Torelli G., Emilia G., Torelli U. (1993). Overexpression of c-kit in a leukemic cell population carrying a trisomy 4 and its relationship with the proliferative capacity. Leuk. Lymphoma.

[33] Miettinen M., Lasota J. (2005). KIT (CD117): A review on expression in normal and neoplastic tissues, and mutations and their clinicopathologic correlation. Appl. Immunohistochem..

[34] Simak R., Capodieci P., Cohen D.W., Fair W.R., Scher H., Melamed J., Drobnjak M., Heston W.D., Stix U., Steiner G. (2000). Expression of c-kit and kit-ligand in benign and malignant prostatic tissues. Histol. Histopathol..

[35] Micke P., Hengstler J.G., Albrecht H., Faldum A., Bittinger F., Becker K., Wiewrodt R., Fischer B., Buhl R. (2004). c-kit Expression in Adenocarcinomas of the Lung. Tumour Biol..

[36] Yasuda A., Sawai H., Takahashi H., Ochi N., Matsuo Y., Funahashi H., Sato M., Okada Y., Takeyama H., Manabe T. (2006). The stem cell factor/c-*kit* receptor pathway enhances proliferation and invasion of pancreatic cancer cells. Mol. Cancer.

[37] McIntyre A., Summersgill B., Grygalewicz B., Gillis A.J., Stoop J., van Gurp R.J., Dennis N., Fisher C., Huddart R., Cooper C. (2005). Amplification and overexpression of the KIT gene Is associated with progression in the seminoma subtype of testicular germ cell tumors of adolescents and adults. Cancer Res..

[38] Smalley K.S.M., Nathanson K.L., Flaherty K.T. (2009). Genetic subgrouping of melanoma reveals new opportunities for targeted therapy. Cancer Sci..

[39] Sakurai S., Fukasawa T., Chong J.M., Tanaka A., Fukayama M. (1999). C-kit gene abnormalities in gastrointestinal stromal tumors (Tumors of Interstitial Cells of Cajal). Cancer Sci..

[40] Sattler M., Salgia R. (2004). Targeting c-Kit mutations: basic science to novel therapies. Leukaemia Res..

[41] Tuveson D.A., Willis N.A., Jacks T., Griffin T.D., Singer S., Fletcher C.D.M., Fletcher J.A., Demetri G.D. (2001). STI571 inactivation of the gastrointestinal stromal tumor c-KIT oncoprotein: biological and clinical implications. Oncogene.

[42] Heinrich M.C., Corless C.L., Blanke C.D., Demetri G.D., Joensuu H., Roberts P.J., Eisenberg B.L., von Mehren M., Fletcher C.D.M., Sandau K. (2006). Molecular correlates of imatinib resistance in gastrointestinal stromal tumors. J. Clin. Oncol..

[43] Rosenzweig S.A. (2012). Acquired resistance to drugs targeting receptor tyrosine kinases. Biochem. Pharmacol..

[44] Bejugam M., Sewitz S., Shirude P.S., Rodriguez R., Shahid R., Balasubramanian S. (2007). Trisubstituted isoalloxazines as a new class of G-Quadruplex binding ligands:  Small molecule regulation of c-kit oncogene expression. J. Am. Chem. Soc..

[45] Ong C.W., Liu M.-C., Lee K.-D., Chang K.W., Yang Y.-T., Tung H.-W., Fox K.R. (2012). Synthesis of bisquinoline–pyrrole oligoamide as G-quadruplex binding ligand. Tetrahedron.

[46] Wang X., Zhou C.-X., Yan J.-W., Hou J.-Q., Chen S.-B., Ou T.-M., Gu L.-Q., Huang Z.-S., Tan J.-H. (2013). Synthesis and evaluation of quinazolone derivatives as a new class of *c*-*KIT* G-Quadruplex binding ligands. ACS Med. Chem. Lett..

[47] Garner T.P., Williams H.E.L., Gluszyk K.I., Roe S., Oldham N.J., Stevens M.F.G., Moses J.E., Searle M.S. (2009). Selectivity of small molecule ligands for parallel and anti-parallel DNA G-quadruplex structures. Org. Biomol. Chem..

[48] Nielsen M.C., Larsen A.F., Abdikadir F.H., Ulven T. (2014). Phenanthroline-2,9-bistriazoles as selective G-quadruplex ligands. Eur. J. Med. Chem..

[49] Gunaratnam M., Swank S., Haider S.M., Galesa K., Reszka A.P., Beltran M., Cuenca F., Fletcher J.A., Neidle S. (2009). Targeting human gastrointestinal stromal tumor cells with a Quadruplex-Binding small molecule. J. Med. Chem..

[50] Cui X., Lin S., Yuan G. (2012). Spectroscopic probing of recognition of the G-quadruplex in c-kit promoter by small-molecule natural products. Int. J. Biol. Macromolec..

[51] Shen F.-H., Jin J., Li J., Wang Y., Zhu S.-H., Lu Y.-J., Ou T.-M., Huang Z.-S., Huang M., Huang Z.-Y. (2013). The G-quadruplex ligand, SYUIQ-FM05, targets proto-oncogene c-*kit* transcription and induces apoptosis in K562 cells. Pharm. Biol..

[52] Chen Z.-F., Qin Q.-P., Qin J.-L., Liu Y.-C., Huang K.-B., Li Y.-L., Meng T., Zhang G.-H., Peng Y., Luo X.-J. (2015). Stabilization of G-Quadruplex DNA, inhibition of telomerase activity, and tumor cell apoptosis by organoplatinum (II) complexes with oxoisoaporphine. J. Med. Chem..

[53] Jantos K., Rodriguez R., Ladame S., Shirude P.S., Balasubramanian S. (2006). Oxazole-based peptide macrocycles:  A new class of G-Quadruplex binding ligands. J. Am. Chem. Soc..

[54] Rahman K.M., Reszka A.P., Gunaratnam M., Haider S.M., Howard P.W., Fox K.R., Neidle S., Thurston D.E. (2009). Biaryl polyamides as a new class of DNA quadruplex-binding ligands. Chem. Commun..

[55] Bejugam M., Gunaratnam M., Müller S., Sanders D.A., Sewitz S., Fletcher J.A., Neidle S., Balasubramanian S. (2010). Targeting the *c-Kit* promoter G-quadruplexes with 6-substituted indenoisoquinolines. ACS Med. Chem. Lett..

[56] Diveshkumar K.V., Sakrikar S., Harikrishna S., Dhamodharan V., Pradeepkumar P.I. (2014). Targeting promoter G-Quadruplex DNAs by indenopyrimidine-Based ligands. Chem. Med. Chem..

[57] McLuckie K.I.E., Waller Z.A.E., Sanders D.A., Alves D., Rodriguez R., Dash J., McKenzie G.J., Venkitaraman A.R., Balasubramanian S. (2011). G-Quadruplex-binding benzo[*a*]phenoxazines down-regulate *c-KIT* expression in human gastric carcinoma cells. J. Am. Chem. Soc..

[58] Diveshkumar K.V., Sakrikar S., Rosu F., Harikrishna S., Gabelica V., Pradeepkumar P.I. (2016). Specific stabilization of *c-MYC* and *c-KIT* G-Quadruplex DNA structures by indolylmethyleneindanone scaffolds. Biochemistry.

[59] Dash J., Nath Das R., Hegde N., Dan Pantos G., Shirude P.S., Balasubramanian S. (2012). Synthesis of bis-indole carboxamides as G-Quadruplex stabilizing and inducing ligands. Chem. Eur. J..

[60] Dhamodharan V., Harikrishna S., Bhasikuttan A.C., Pradeepkumar P.I. (2015). Topology specific stabilization of promoter over telomeric G-Quadruplex DNAs by bisbenzimidazole carboxamide derivatives. ACS Chem. Biol..

[61] Wei C., Ren L., Gao N. (2013). Interactions of terpyridines and their Pt(II) complexes with G-quadruplex DNAs and telomerase inhibition. Int. J. Biol. Macromol..

[62] Waller Z.A.E., Sewitz S.A., Hsu S.-T.D., Balasubramanian S. (2009). A small molecule that disrupts G-Quadruplex DNA structure and enhances gene expression. J. Am. Chem. Soc..

[63] Manaye S., Eritja R., Aviñó A., Jaumot J., Gargallo R. (2012). Porphyrin binding mechanism is altered by protonation at the loops in G-quadruplex DNA formed near the transcriptional activation site of the human *c-kit* gene. Biochim. Biophys. Acta.

[64] Petenzi M., Verga D., Largy E., Hamon F., Doria F., Teulade-Fichou M.-P., Guédin A., Mergny J.-L., Mella M., Freccero M. (2012). Cationic pentaheteroaryls as selective G-Quadruplex ligands by solvent-free microwave-assisted synthesis. Chem. Eur. J..

[65] Alzeer J., Luedtke N.W. (2010). PH-Mediated fluorescence and G-Quadruplex binding of amido phthalocyanines. Biochemistry.

[66] Zheng K.-W., Zhang D., Zhang L.-X., Hao Y.-H., Zhou X., Tan Z. (2011). Dissecting the strand folding orientation and formation of G-Quadruplexes in single- and double-stranded nucleic acids by ligand-induced photocleavage footprinting. J. Am. Chem. Soc..

[67] Marchetti C., Minarini A., Tumiatti V., Moraca F., Parrotta L., Alcaro S., Rigo R., Sissi C., Gunaratnam M., Ohnmacht S.A. (2015). Macrocyclic naphthalene diimides as G-quadruplex binders. Bioorg. Med. Chem..

[68] Islam M.M, Fujii S., Sato S., Okauchi T., Takenaka S. (2015). A selective G-Quadruplex DNA-stabilizing ligand based on a cyclic naphthalene diimide derivative. Molecules.

[69] Zorzan E., Da Ros S., Musetti C., Shahidian L.Z., Coelho N.F.R., Bonsembiante F., Létard S., Gelain M.E., Palumbo M., Dubreuil P. (2016). Screening of candidate G-quadruplex ligands for the human *c-KIT* promotorial region and their effects in multiple in-vitro models. Oncoterget.

[70] Głuszyńska A. (2015). Biological potential of carbazole derivatives. Eur. J. Med. Chem..

[71] Głuszyńska A., Juskowiak B., Kuta-Siejkowska M., Hoffmann M., Haider S. (2018). Carbazole ligands as c-myc G-quadruplex binders. Int. J. Biol. Macromol..

[72] Głuszyńska A., Bajor K., Czerwińska I., Kalet D., Juskowiak B. (2010). The synthesis and spectral properties of new DNA binding ligands. Tetrahedron Lett..

[73] Głuszyńska A., Rajczak E., Juskowiak B. The synthesis and spectral properties of new carbazole ligand, potential DNA intercalator. Proceedings of the 40th International Conference of Slovak Society of Chemical Engineering.

[74] Głuszyńska A., Rajczak E., Juskowiak B. (2013). Synthesis and spectroscopic characterisation of (*E*)-2-(2-(9-(4-(1*H*-1,2,4-triazol-1-yl)butyl)-9*H*-carbazol-3-yl)vinyl)-3-ethylbenzo[*d*]thiazol-3-ium, a new ligand and potential DNA intercalator. Chem. Pap..

[75] Głuszyńska A., Rajczak E., Kosman J., Juskowiak B. Interactions of New Carbazole Ligands, Potential Inhibitors of Telomerase, with Different Structures of DNA: A Comparative Study. Proceedings of the 40th International Conference of Slovak Society of Chemical Engineering.

[76] Głuszyńska A., Burzyńska K., Rajczak E., Juskowiak B. Tatranské Matliare, Slovakia. Interaction of N-phenyl carbazole Ligands with Double Stranded structures of DNA. Proceedings of the 40th International Conference of Slovak Society of Chemical Engineering.

[77] Saengkhae C., Salerno M., Adès D., Siove A., Le Moyec L., Migonney V., Garnier-Suillerot A. (2007). Ability of carbazole salts, inhibitors of Alzheimer β-amyloid fibril formation, to cross cellular membranes. Eur. J. Pharmacol..

[78] Głuszyńska A., Kowal A., Kolasa A., Rajczak E., Juskowiak B., Rubiś B., Takenaka S. Synthesis and characterization of fluorescent DNA binding carbazole ligand.

[79] Phan A.T., Kuryavyi V., Luu K.N., Patel D.J., Neidle S., Balasubramanian S. (2006). Quadruplex Nucleic Acids.

[80] Bhattacharjee A.J., Ahluwalia K., Taylor S., Jin O., Nicoludis J.M., Buscaglia R., Chaires J.B., Kornfilt D.J.P., Marquardt D.G.S., Yatsunyk L.A. (2011). Induction of G-quadruplex DNA structure by Zn (II) 5,10,15,20-tetrakis (*N*-methyl-4-pyridyl)porphyrin. Biochimie.

[81] Yamashita T., Uno T., Ishikawa Y. (2005). Stabilization of guanine quadruplex DNA by the binding of porphyrins with cationic side arms. Bioorg. Med. Chem..

[82] Shi S., Liu J., Li J., Zheng K.-C., Huang X.-M., Tan C.-P., Chen L.-M., Ji L.-N. (2006). Synthesis, characterization and DNA-binding of novel chiral complexes D- and K-[Ru(bpy)2L] ^2+^ (L = o-mopip and p-mopip). J. Inorg. Biochem..

[83] Sun J., An Y., Zhang L., Chen H.-Y., Han Y., Wang Y.-J., Mao Z.-W., Ji L.-N. (2011). Studies on synthesis, characterization, and G-quadruplex binding of Ru (II) complexes containing two dppz ligands. J. Inorg. Biochem..

[84] Maji B., Kumar K., Kaulage M., Muniyappa K., Bhattacharya S. (2014). Design and synthesis of new benzimidazole−carbazole conjugates for the stabilization of human telomeric DNA, telomerase inhibition, and their selective action on cancer cells. J. Med. Chem..

[85] Maji B., Kumar K., Muniyappa K., Bhattacharya S. (2015). New dimeric carbazole–benzimidazole mixed ligands for the stabilization of human telomeric G-quadruplex DNA and as telomerase inhibitors. A remarkable influence of the spacer. Org. Biomol. Chem..

[86] Masiero S., Trotta R., Pieraccini S., De Tito S., Perone R., Randazzo A., Spada G.P. (2010). A non-empirical chromophoric interpretation of CD spectra of DNA G-quadruplex structures. Org. Biomol. Chem.,.

[87] Fan J.-H., Bochkareva E., Bochkarev A., Gray D.M. (2009). Circular dichroism spectra and electrophoretic mobility shift assays show that human replication protein a binds and melts intramolecular G-Quadruplex structures. Biochemistry.

[88] Crosby G.A., Demas J.N. (1971). Measurement of photoluminescence quantum yields. Review. J. Phys. Chem..

[89] Mergny J.-L., Phan A.-T., Lacroix L. (1998). Following G-quartet formation by UV-spectroscopy. FEBS Lett..

[90] Mergny J.-L., Lacroix L. (2003). Analysis of thermal melting curves. Oligonucleotides.

[91] Rachwal P.A., Fox K.R. (2007). Quadruplex melting. Methods.

[92] Tataurov A.V., You Y., Owczarzy R. (2008). Predicting ultraviolet spectrum of single stranded and double stranded deoxyribonucleic acids. Biophys. Chem..

[93] Wang J., Wolf R.M., Caldwell J.W., Kollman P.A., Case D.A. (2004). Development and testing of a general amber force field. J. Comput. Chem..

[94] Hornak V., Abel R., Okur A., Strockbine B., Roitberg A., Simmerling C. (2006). Comparison of multiple amber force fields and development of improved protein backbone parameters. Proteins.

[95] Pérez A., Marchán I., Svozil D., Sponer J., Cheatham T.E., Laughton C.A., Orozco M. (2007). Refinement of the AMBER force field for nucleic acids: improving the description of α/γ conformers. Biophys. J..

[96] Zgarbová M., Otyepka M., Šponer J., Mládek A., Banáš P., Cheatham T.E., Jurečka P. (2011). Refinement of the cornell et al. nucleic acids force field based on reference quantum chemical calculations of glycosidic torsion profiles. J. Chem. Theory Comput..

[97] Banáš P., Hollas D., Zgarbová M., Jurečka P., Orozco M., Cheatham T.E., Šponer J., Otyepka M. (2010). Performance of molecular mechanics force fields for RNA simulations: stability of UUCG and GNRA hairpins. J. Chem. Theory Comput..

[98] Case D.A., Cheatham T.E., Darden T., Gohlke H., Luo R., Merz K.M., Onufriev A., Simmerling C., Wang B., Woods R.J. (2005). The Amber biomolecular simulation programs. J. Comput. Chem..

[99] Abagyan R., Totrov M., Kuznetsov D. (1994). ICM—A new method for protein modeling and design: Applications to docking and structure prediction from the distorted native conformation. J. Comput. Chem..

[100] Harvey M.J., Giupponi G., Fabritiis G.D. (2009). ACEMD: Accelerating biomolecular dynamics in the microsecond time Scale. J. Chem. Theory Comput..

[101] Van Der Spoel D., Lindahl E., Hess B., Groenhof G., Mark A.E., Berendsen H.J.C. (2005). Gromacs: Fast, flexible, and free. J. Comput. Chem..

[102] Humphrey W., Dalke A., Schulten K. (1996). VMD: Visual molecular dynamics. J. Mol. Graph..

[103] Humphrey W., Dalke A., Schulten K. (1996). VMD: Visual molecular dynamics. J. Mol. Graph..

[104] Maiorov V.N., Crippen G.M. (2004). Size-independent comparison of protein three-dimensional structures. Proteins.

