# Supplementary material for: Carbazole Derivatives’ Binding to c-KIT G-Quadruplex DNA
Source: Molecules. 2018 May 10;23(5):1134. doi: 10.3390/molecules23051134 (PMC6099540; doi:10.3390/molecules23051134)
Supplement: Supplementary file 1 [file molecules-23-01134-s001.pdf]

Supplementary Materials

Carbazole Derivatives' Binding to c-KIT G-quadruplex DNA

Agata Głuszyńska [1,*], Bernard Juskowiak [1], Martyna Kuta-Siejkowska [2], Marcin Hoffmann [2] and Shozeb Haider [3]

1 Laboratory of Bioanalytical Chemistry, Faculty of Chemistry, Adam Mickiewicz University, Poznań 61–614, Umultowska Street 89b, Poland; juskowia@amu.edu.pl
2 Laboratory of Quantum Chemistry, Faculty of Chemistry, Adam Mickiewicz University, Poznań 61–614, Umultowska Street 89b, Poland; martyna.kuta@o2.pl (M.K.-S.), mmh@amu.edu.pl (M.H.)
3 School of Pharmacy, University College London, London WC1N 1AX, UK; shozeb.haider@ucl.ac.uk
* Correspondence: aglusz@amu.edu.pl; Tel.: +48-618-291-1770

Contents

Fig. S1. Spectrophotometric titration of ligands **2** (A), **3** (B) with G4 c-KIT 2O3M.

Fig. S2. Fluorescence titration spectra of ligands **1** (A), **2** (B) with G4 c-KIT 2O3M.

Fig. S3. Benesi-Hildebrand plots for absorbance binding data of ligands with G4 c-KIT 2O3M.

Fig. S4. Benesi-Hildebrand plots for fluorescence binding data of ligands with G4 c-KIT 2O3M.

Figs. S5-S16 Molecular modeling results.

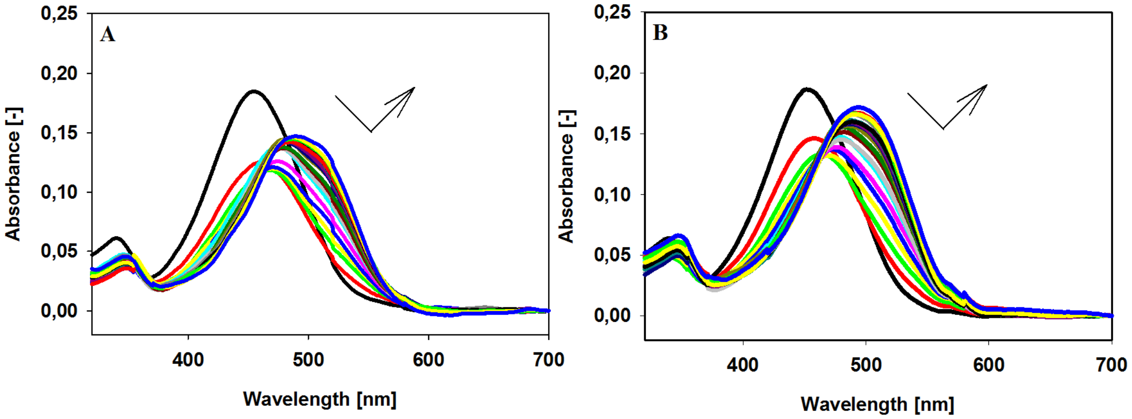

Figure S1. Spectrophotometric titration of ligands **1** (A), **3** (B) (6 μM) with G4 c-KIT 2O3M (0 - 30 μM) in Tris-HCl buffer (10 mM, pH 7.2) containing 100 mM KCl.

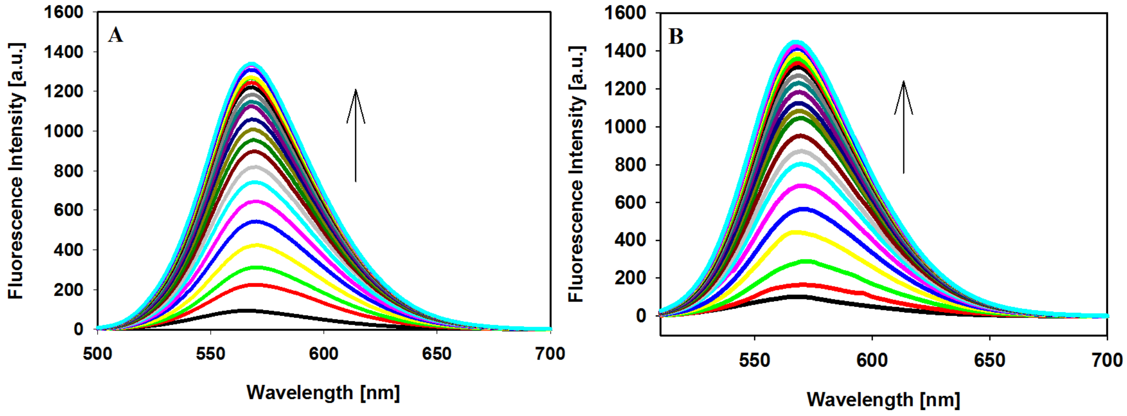

Figure S2. Fluorescence titration spectra of ligands **2** (A), **3** (B) (2 µM) with G4 c-KIT 2O3M (0 - 30 µM) in Tris-HCl buffer (10 mM, pH 7.2) containing 100 mM KCl; λ_{ex}: **2** - 492 nm, **3** - 493 nm.

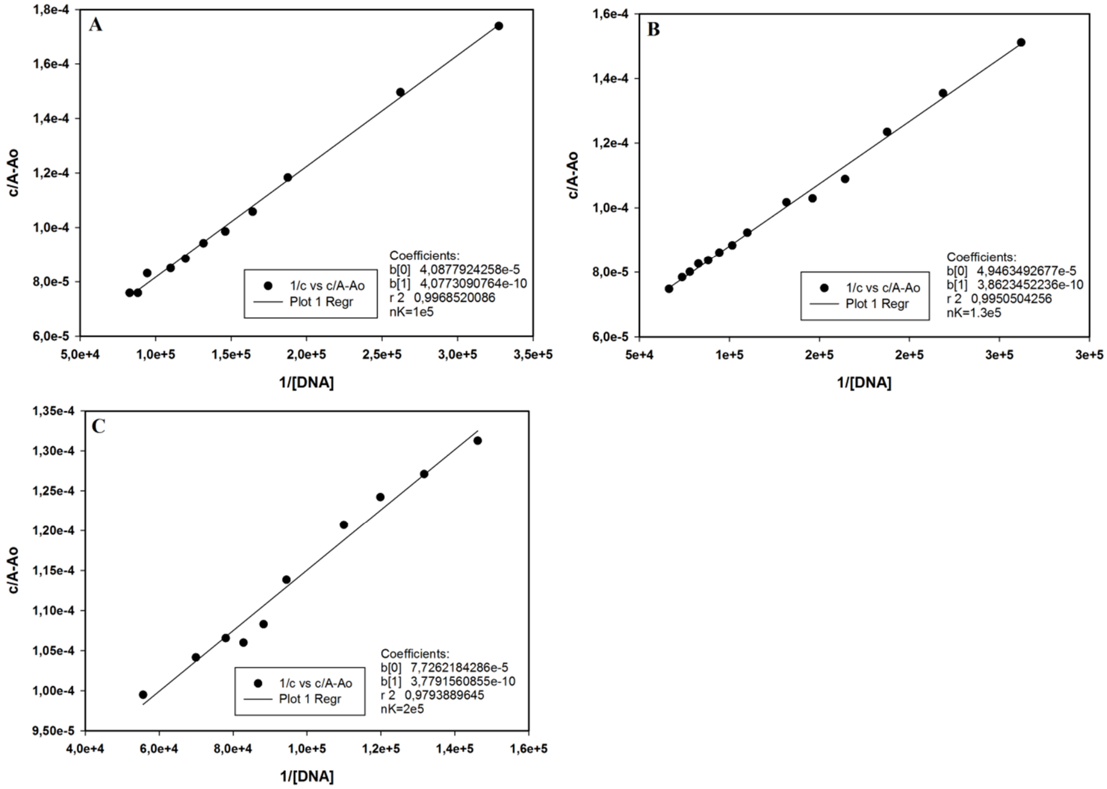

Figure S3. Benesi-Hildebrand plots of absorbance binding data of ligands **1** (A), **2** (B), and **3** (C) with G4 2O3M.

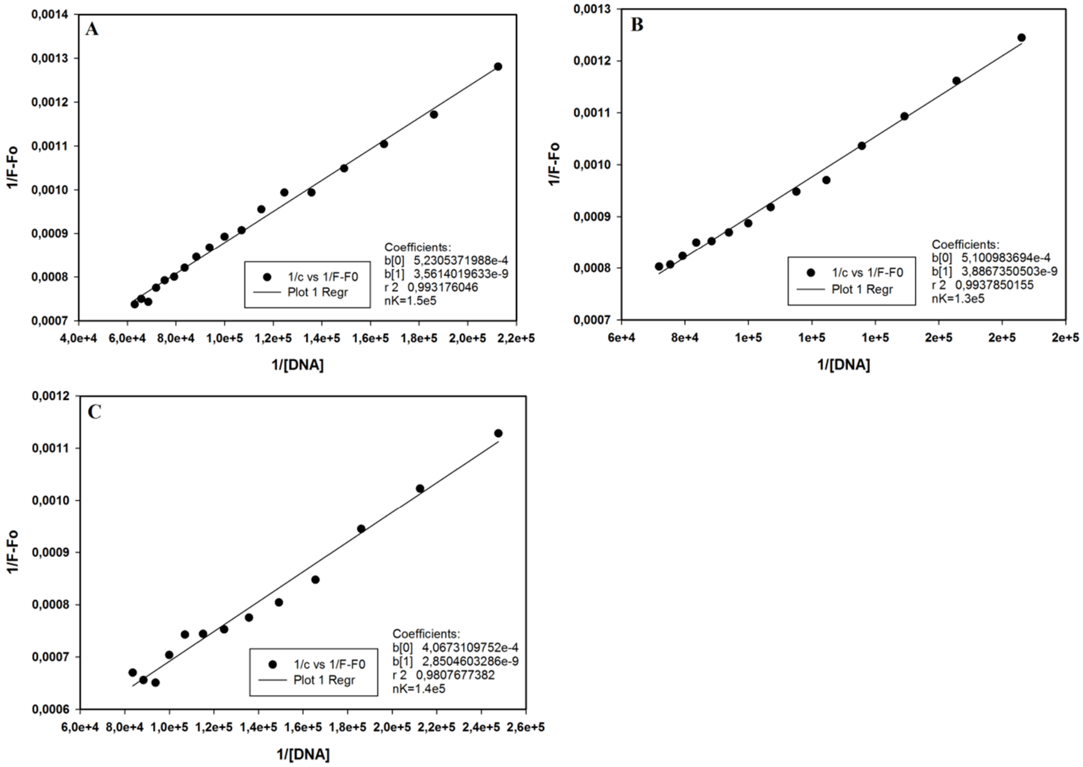

Figure S4. Benesi-Hildebrand plots of fluorescence binding data of ligands **1** (A), **2** (B) and **3** (C) with G4 2O3M.

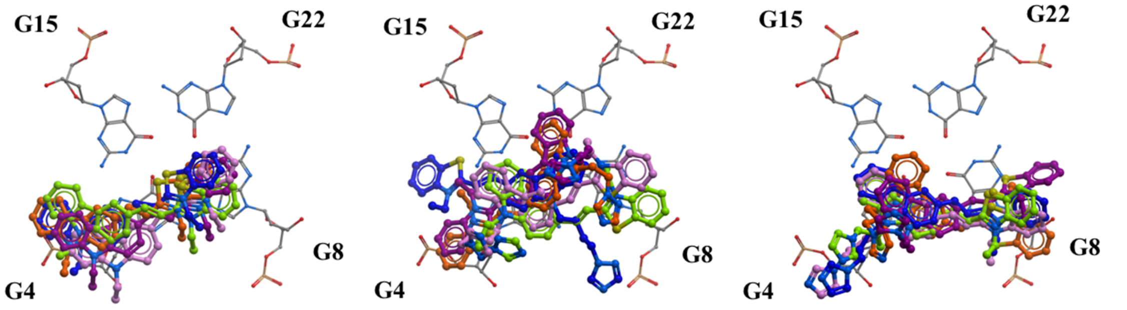

Figure S5. Aligned orientations of ligands **1 (A)**, **2 (B)**, **3 (C)**, positioned above G-tetrad, extracted from the simulations at every 200 ns. Pictures represents 3'end of G-quadruplex. Colour of the ligands denotes the step number. Step 1 – 200ns (green), step 2 - 400ns (pink), step 3 - 600ns (blue), step 4 – 800ns (orange), step 5 – 1000ns (magneta).

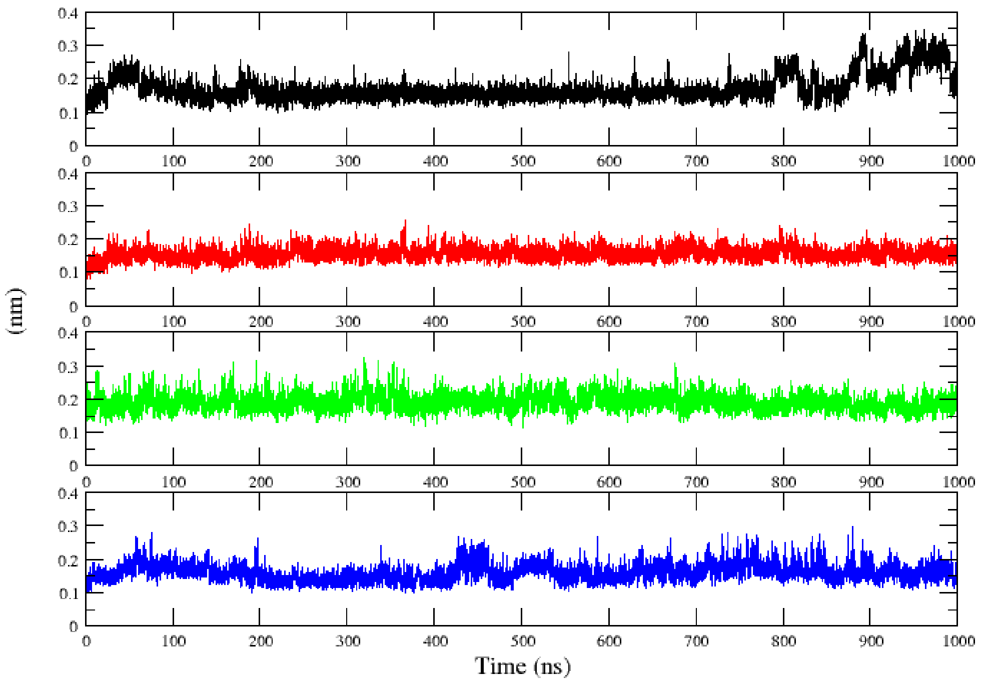

Figure S6. Plot of RMSD of G-quadruplex backbone atoms calculated along the 1000ns simulation time. The figure illustrates RMSD values in complex with ligand 1 (red), 2 (green), 3 (blue) and for the native G-quadruplex (black).

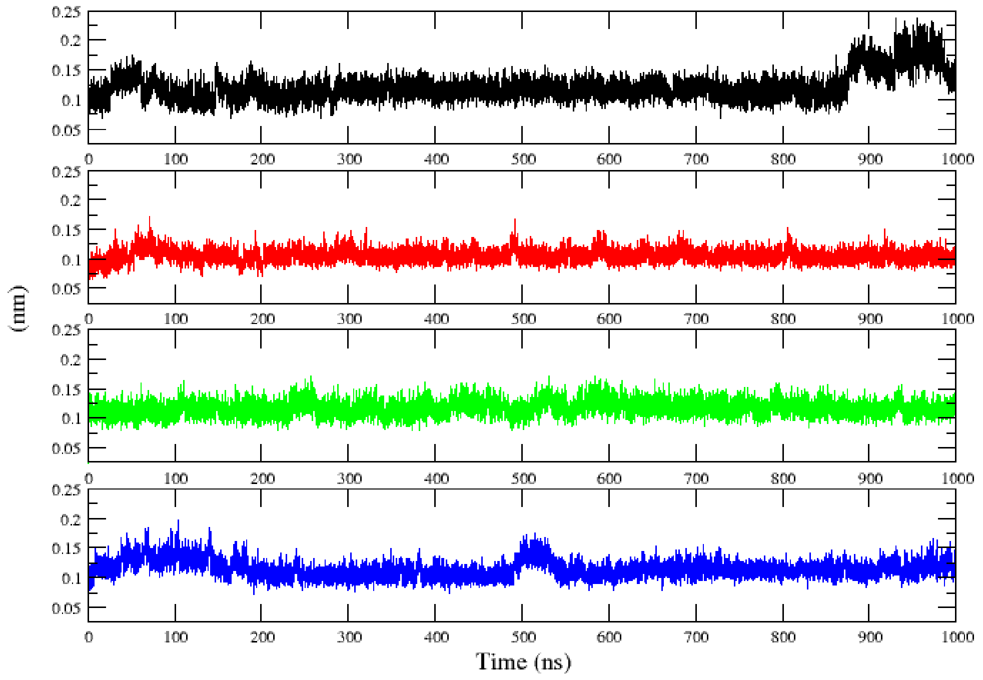

Figure S7. Plot of RMSD of G-quadruplex tetrad atoms calculated along the 1000ns simulation time. The figure illustrates RMSD values in complex with ligand 1 (red), 2 (green), 3 (blue) and for the native G-quadruplex (black).

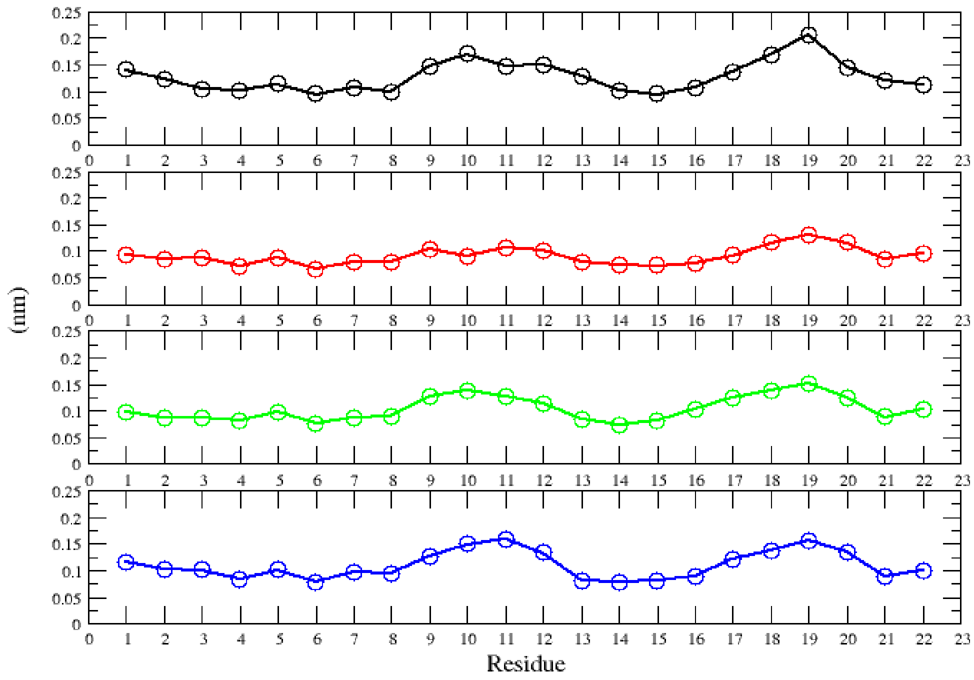

Figure S8. RMSF of G-quadruplex backbone atoms calculated along the 1000ns simulation time. The figure illustrates RMSF values in complex with ligand 1 (red), 2 (green), 3 (blue) and for the native G-quadruplex (black).

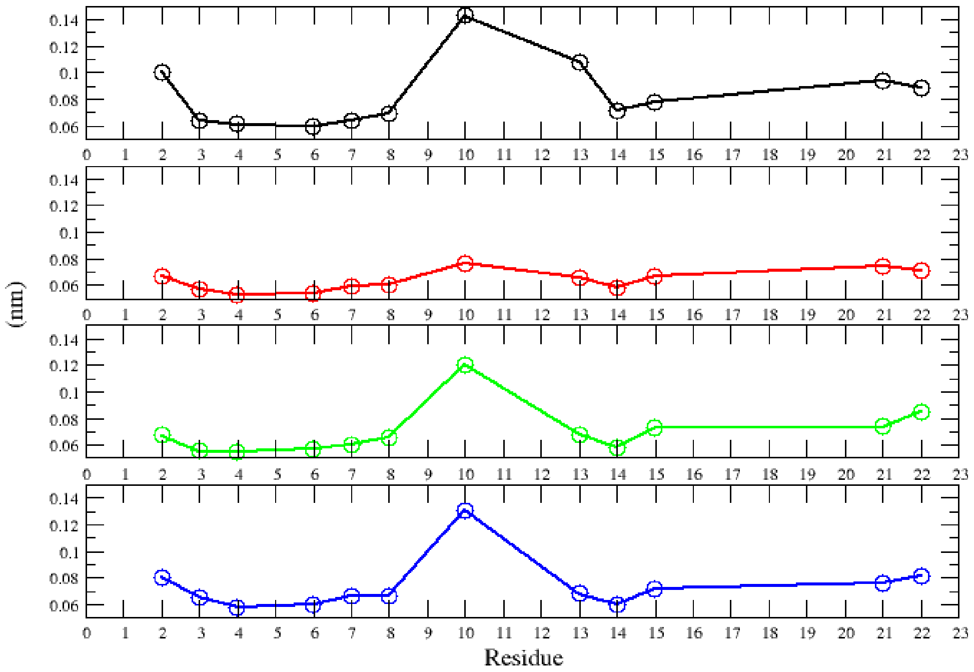

Figure S9. RMSF of G-quadruplex tetrad atoms calculated along the 1000ns simulation time. The figure illustrates RMSF values in complex with ligand 1 (red), 2 (green), 3 (blue) and for the native G-quadruplex (black).

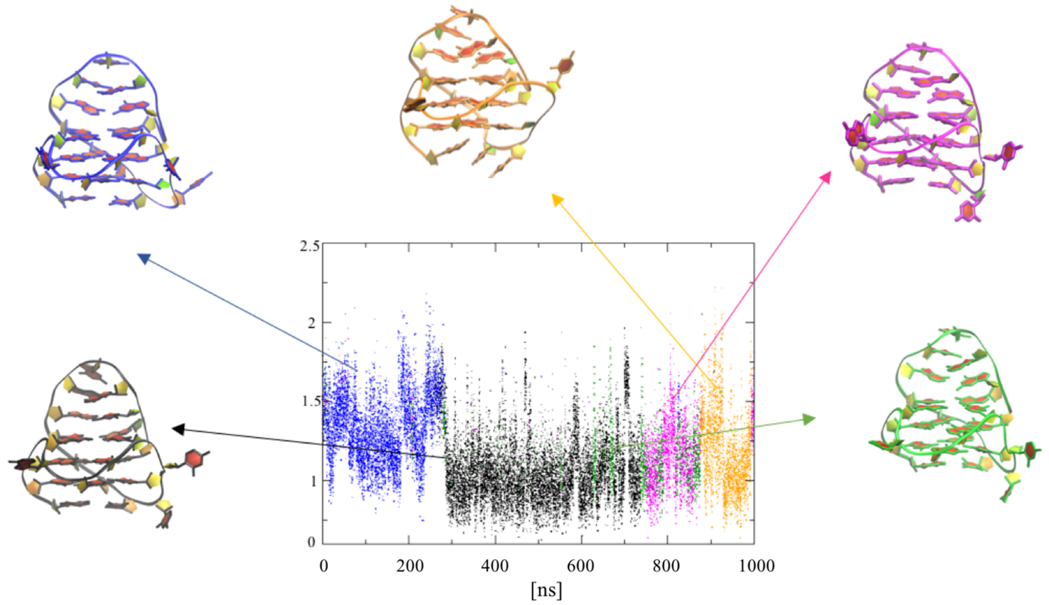

Figure S10. Representation of the conformational clusters obtained by clustering analysis for simulated DNA without ligand. RMSD-based clustering, with a cut-off of 2.3 Å, through trajectory identified five clusters. Cartoon representations of side view of cluster centers are illustrated (top 3' end). A 2.3 Å RMSD cut-off identified 5 clusters for DNA conformations simulated without ligand (Fig. S10). The first 300 ns is represented by the blue centroid, which comprised of around 26% of the entire simulation. The largest cluster (black) makes up of 43 % of the entire simulation. Then conformation is slightly changing through green (7.3 % of the entire simulation) to pink and then orange centroid (both comprised around 11.5 % of entire simulation) representation of the G4 conformation. The most significant differences between clusters are observed for loop C9-G10-C11 and residue A5 (Fig. S11.)

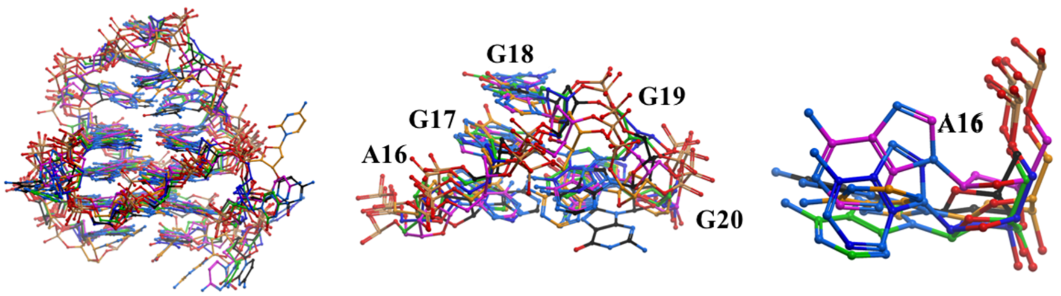

Figure S11. Representation of alignment of the centroids from cluster 1 (black), 2 (blue), 3 (orange), 4 (magenta) and 5 (green) for DNA after simulations without ligand.

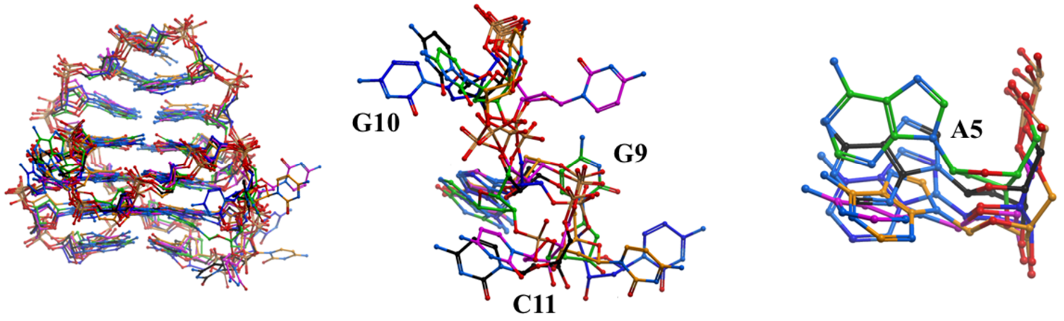

Figure S12. Representation of alignment of top centroid from cluster 1 (black), 2 (blue), 3 (orange), 4 (magenta) and 5 (green)for DNA after simulations with ligand **3**.

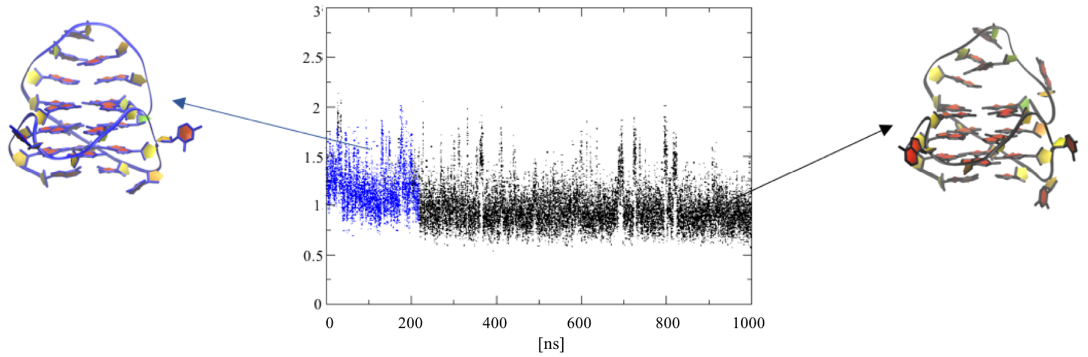

Figure S13. Representation of the conformational clusters obtained by clustering analysis for DNA simulated with ligand **1**. RMSD-based clustering, with a cut-off of 2.3 Å, through trajectory identified three clusters. Cartoon representations of side view of cluster centres are illustrated (top 3' end).

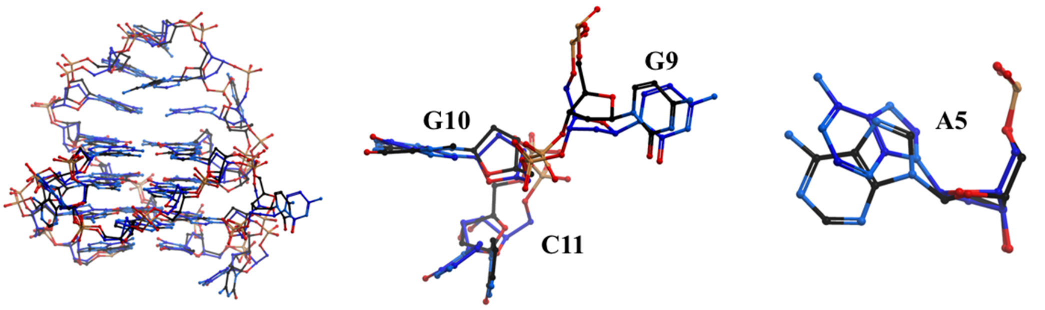

Figure S14. Representation of alignment of centroids from cluster 1 (black) and 2 (blue) for DNA after simulations with ligand **1.**

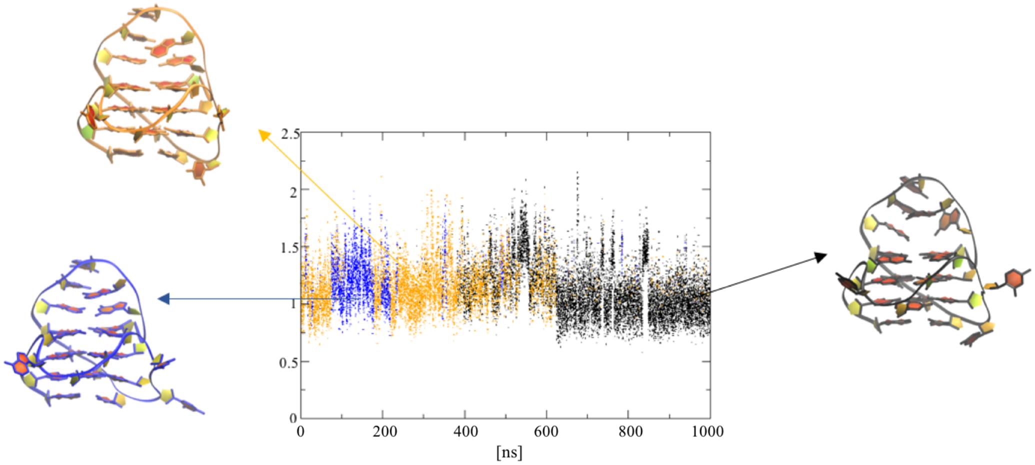

Figure S15. Representation of the conformational clusters obtained by clustering analysis for DNA simulated with ligand **2**. RMSD-based clustering, with a cut-off of 2.3 Å, through trajectory identified three clusters. Cartoon representations of side view of cluster centres are illustrated (top 3' end).

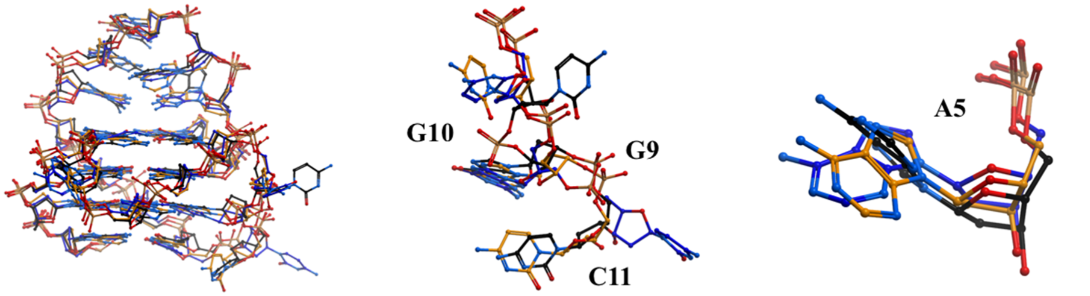

Figure S16. Representation of alignment of the centroids from cluster 1 (black), 2 (blue) and 3 (orange) for DNA after simulations with ligand **2**.
